# Media use, attention, mental health and academic performance among 8 to 12 year old children

**DOI:** 10.1371/journal.pone.0259163

**Published:** 2021-11-17

**Authors:** Pedro Cardoso-Leite, Albert Buchard, Isabel Tissieres, Dominic Mussack, Daphne Bavelier

**Affiliations:** 1 University of Luxembourg, Department of Behavioral and Cognitive Science, Esch-sur-Alzette, Luxembourg; 2 Université de Genève, Faculté de Psychologie et Sciences de l’Education (FPSE), Geneva, Switzerland; 3 Campus Biotech, Geneva, Switzerland; Birkbeck College, UNITED KINGDOM

## Abstract

The rise in digital media consumption, especially among children, raises the societal question of its impact on cognition, mental health and academic achievement. Here, we investigate three different ways of measuring technology use-—total hours of media consumed, hours of video game play and number of media used concurrently—-in 118 eight-to-twelve year-old children. At stake is the question of whether different technology uses have different effects, which could explain some of the past mixed findings. We collected data about children’s media uses as well as (i) attentional and behavioral control abilities, (ii) psychological distress, psychosocial functioning, and sleep, and (iii) academic achievement and motivation. While attentional control abilities were assessed using both cognitive tests and questionnaires, mental health and sleep were all questionnaire-based. Finally, academic performance was based on self-reported grades, with motivational variables being measured through the grit and the growth-mindset questionnaires. We present partial correlation analyses and construct a psychological network to assess the structural associations between different forms of media consumption and the three categories of measures. We observe that children consume large amounts of media and media multitask substantially. Partial correlation analyses show that media multitasking specifically was mostly correlated with negative mental health, while playing video games was associated with faster responding and better mental health. No significant partial correlations were observed for total hours on media. Psychological network analysis complement these first results by indicating that all three ways of consuming technology are only indirectly related to self-reported grades. Thus, technology uses appear to only indirectly relate to academic performance, while more directly affecting mental health. This work emphasizes the need to differentiate among technology uses if one is to understand how every day digital consumption impacts human behavior.

## Introduction

Digital media consumption (e.g., watching videos, listening to music, playing video games) has increased drastically over the past decades. In the US, 8–12 year old children spend an average of almost 6 hours on digital media every single day [1] with a substantial fraction of that time spent on multiple media at the same time [29% for 7th to 12th graders; [2]]. In the European Union, 10–14 year olds spend an average of 2.8 hours a day on digital screens; for 15–19 year olds that number increases to about 3 hours per day [3]. These numbers reflect the ubiquitous role that digital media has come to play in our lives, a role that is very likely to keep growing both in terms of the magnitude and the diversity of digital media consumption. This state of affairs raises increasing concerns about the impact of digital media consumption, in particular among children.

The emerging literature on the impact of media use on cognition paints a rather complex picture whereby different media have distinct, and possibly opposite effects. For example video games have been shown to have different effects on various cognitive dimensions depending on the specific game genre played [4, 5]. Children’s media consumption is frequently assessed through total time on media as if all forms of media represented a unitary experience. Yet, this is clearly not the case [6]. Research increasingly suggests that the impact of digital media use on cognition, academic performance or health is complex [e.g., 7, 8], as it depends on the type of media (e.g., video games, social networks), its content (e.g., fantasy, documentary), the context (e.g., alone, in groups) and the traits of the person consuming media [e.g., age, gender]. To understand how digital media impacts children’s attentional and behavioral control, mental health and academic achievement requires a finer grained approach [9–11].

The present work builds on this recognized need for a finer grained approach. First it presents new experimental data which takes a more granular approach to media consumption by investigating three media consumption indices—-total time on media, media multitasking and video game play. Second, this work assesses the relationships of these forms of media consumption with children’s cognition as measured not only through surveys but also task-based measures of attentional and behavioral control; the latter providing finer measures of cognition than surveys. In addition, mental health, and school related variables were also collected on the same children, allowing us to evaluate the relative association strength of different media consumption types on different aspects of children’s lives. The third contribution of this work is methodological. Studying the impact of media on humans poses a number of challenges: many of the variables considered in this research field correlate with each other (e.g., total hours on media correlates with the amount of media multitasking) and there are yet no clear, established causal models in this emerging field (e.g., does multitasking increase impulsivity or do impulsive people multitask more?). We apply psychological networks—a relatively new modeling technique—and discuss how it provides valuable insights that complement the more traditional pairwise correlations, linear regressions or mediation analyses.

### Previous studies

The current cognitive literature suggests that different forms of media consumption have different relationships and possibly casual effects on human cognition. In particular, while playing action video games has been linked to enhanced cognition, and in particular attentional control, media multitasking has been linked to worse attentional control, greater distractibility or attention lapses. Such contrasting relationships also call into question the rationale of considering all screen times in an undifferentiated way as is common in the literature. This work therefore focuses on three measures of media usage: total time on media, media multitasking and video gaming. We review in turn their respective relationships with cognition, mental health and a few school related variables, among which academic performance.

#### Total time on media

Many studies report that total time on media (all types of media or all screen based media) is associated with adverse *attentional and behavioral* outcomes [e.g., 12–15] and in particular Attention-Deficit/Hyperactivity Disorder (ADHD) symptoms. A two year longitudinal study on more than three thousand 15–16 year olds reported that a higher frequency of media use at baseline was associated with a subsequent increase in ADHD symptoms [16]. Similarly, a meta-analysis on the relation between total time on media and ADHD-related behaviors reported a small but significant association [17], as measured by either cognitive tasks, surveys, or observations.

Total time on media has also been linked to various *mental health* problems. Limtrakul et al. [18], for example, explored the relationships between self-reported media use variables and psychosocial health—as measured by the Strengths and Difficulties Questionnaire [19] which has been linked to Problematic Media Use [20] among 10–15 year olds. Their results suggest that larger amounts of total time spent on media is associated with lower prosocial behaviour scores (measured with items like: the child is “kind to younger children”). More time on digital technology has also been linked to decreased well-being in adolescents. Indeed, Orben & Przybylski (2019) documented such a negative tendency using several large-scale datasets [21]. Yet, these authors also stressed that this relationship is so small as to be negligible.

The relationship between total time on media and *school related variables* is less clear, with large-scale studies reporting somewhat contradictory results [e.g., 22, 23]. A recent meta-analysis on the relationship between overall “screen” media use (i.e., computer, internet, mobile phone, television, video game) and academic performance among children and adolescents (4–18 year olds) reported no relationship between total time on digital media and academic performance [7]. This same study however, reported that both larger amounts of time spent watching television or playing video games were associated with lower academic performance.

#### Media multitasking

Media multitasking, the simultaneous use of multiple digital media (e.g., listening to music while surfing the web), is both an expanding, recent societal phenomenon [1, 24, 25] and an active research topic since the seminal study by Ophir et al. [26] reported that young adults who media multitask heavily exhibited impairments in suppressing distractions across multiple cognitive tests.

Media multitasking has been associated with a broad range of cognitive impairments [27–30], most notably in *attentional and behavioral* control—in particular top-down control of attention, such as inhibiting distractions or avoiding attention lapses, and behavioral control such as avoiding impulsive behavior. Indeed, media multitasking has been associated with higher scores on ADHD surveys [31–33], and higher levels of impulsivity [32, 34–36] and mind-wandering [37], but see [38]—-in line with these results, Kobayashi et al. [39] reported differences in functional connectivity of the dorsal attentional network when comparing heavy and light media multitaskers. Furthermore, Ophir et al. [26] reported that heavy media multitaskers performed worse than light media multitaskers on a range of cognitive tasks, including working memory, task switching and selective attention tasks. A recent neuroimaging study further points to altered memory retrieval in high media multitaskers, owing to more frequent attentional lapses during the processing of memory retrieval cues [40]. Other studies did not always replicate these results [34, 41–43] or suggested that the relationships between levels of media multitasking and cognitive performance may be non-linear [44, 45]. The results seem clearer when using surveys and self-reports rather than computerized tests [31]: media multitasking has been associated with deficits in self-reported everyday executive and attentional functions [31, 37, 46, 47] and could be particularly detrimental at younger ages where executive functions still develop [48]; see also [49].

Media multitasking has also been associated with *mental health* problems. Becker et al. [50] for instance, reported that media multitasking was positively correlated with depression and social anxiety scores, even after controlling for total time on media and personality traits. High levels of media multitasking have been linked to less sleep, difficulties to fall asleep at night and to keep awake during the day, at school [51, 52]. However, a longitudinal study found no temporal association between media multitasking and sleep [53], suggesting that the relationship between media multitasking and sleep might not be a direct causal one.

Finally, media multitasking has been linked to negative academic performance and other *school related variables*. Some studies for instance report that heavy media multitaskers are less efficient academic learners [54] and may have less grit [50]—-the ability to maintain perseverance in otherwise aversive tasks, which seems important for academic success [55]. Cain et al. [56] studied 12–16 year olds and reported that heavy media multitasking was associated with lower academic performance on standardized tests (Math and English) but also with lower performance on computerized executive functions tests and higher impulsivity, along with lesser growth mindset (but neither grit nor conscientiousness, in contrast to other studies mentioned), suggesting that media multitasking is a critical variable to consider when investigating the effects of media [see also 57, 58].

#### Video game play

Several meta-analyses document a positive impact of specifically action video games (AVG)–as compared to other types of video games–on cognition [e.g., 59–63, but see 64]. Playing AVG has been frequently related to improved *attentional control* and in particular improved top-down (but not bottom-up) attention [63]. AVG, defined in this literature as those in the first or third-person shooter genres, appear to have a greater positive impact on cognition than other types of video games [63]. In cross-sectional studies of attentional control on 7–22 year old participants who were classified as either being AVG players or non-video game players, [65] observed systematic attentional advantages in the AVG players group. These results are further supported by a few intervention studies on children. For example, Franceschini et al. [66, 67] trained 7–13 years old dyslexic children using various mini-games for 12 hours, distributed over multiple days. The experimental group played mini-games that used action video game mechanics while the control group played mini-games that did not share those features [4, for a discussion of action game features, see 68]. The results showed an improvement in attention (and in reading) only for the experimental group that trained with action-like mini-games. The positive relationship between action video game and cognition is unlikely to hold for video games at large. Indeed, the bulk of intervention studies using action video games makes it clear that not all video games have the same impact on cognition.

The relationship between video game play and *mental health* are somewhat mixed. A large-scale study (N = 2442), on 7–11 year old children for instance, reported that large amounts of gaming (more than 9 hours per week)—but not smaller amounts—were associated with increased conduct problems and reduced prosocial behavior [69]. Similar conclusions seem to hold in older children [10-15 year-olds; [70]]: compared to children who do not play video games at all, children who play daily for more than 3 hours presented less prosocial behaviors, more conduct problems and decreased life-satisfaction. Children who played between 1 and 3 hours per day were equivalent in those measures as children who did not play at all. Surprisingly however, playing less than 1 hour per day was linked to the opposite pattern of results, suggesting that small amounts of video gaming might in fact have positive effects [70]. This hypothesis is corroborated by a European study on more than three thousand 6–11 year-olds [71] which reported no sign of increased mental health problems as a function of video game play and instead, suggested that gaming might have a protective effect against difficult social relationships.

Finally, there have also been mixed results on the relationship between playing video games and *school related variables*. After correcting for multiple demographic and trait-level variables, larger amounts of video gaming was linked with greater intellectual functioning and school achievement (as rated by the child’s teacher) relative to other children in the class [71]. Similarly, Pujol et al. [69] found a positive association between game play and the teacher’s rating of school achievement, but no trend with the number of hours played [see also 72]. The relationship between video gaming and academic performance remains, however, unclear [73]: it can be positive, negative or absent depending on various factors (e.g., playing during weekdays versus weekend days [74]; playing before versus after school [75]). The relationship between gaming and academic outcomes might be U-shaped rather than monotonic, and might for instance depend on the type of games played [76]. There are some reasons to believe that action video games in particular might benefit educational outcomes [66, e.g., 77, 78].

### The present study

The reviewed literature shows that different forms of media consumption may affect attentional/behavioral control, mental health and school related variables in different ways. Given the ubiquity of digital media in our lives and the concern that their potential adverse effects may be amplified in younger children, it is imperative to further our understanding of these effects and how they relate to each other.

The reviewed literature is filled with hypotheses about the potential causal relationships between any two constructs, but oftentimes the data to support specific claims is simply missing [79]. It is unclear, for example, why exactly total time on media should correlate with attentional/behavioral control, mental health or school related variables. Total time on media is a rough measure of media consumption and there are potentially many confounding variables (e.g., media multitasking habits) which might be responsible for the observed associations. Furthermore, total time on media might have only an indirect effect. For instance, total time on media may affect attention, mental health and school related variables via its negative effects on sleep [80], which might be the real cause of decreased cognitive functioning, mental health and academic performance [e.g., 81, 82]. It appears then, that in order to gain insights into the underlying relationships it is necessary to collect for each participant a larger set of measures covering both different aspects of their media use habits, but also aspects of their cognitive functioning, their mental health and, in the case of children, school related variables. This insight motivated the design of the present study. Note that cross-sectional data (as reported here and in most of the cited literature) is fundamentally limited in its ability to support causal claims; specific causal relationships are best established within intervention studies.

The second important insight is methodological and concerns how to best analyse such multivariate data. Previous cross-sectional research mostly used correlation and linear regression analyses to highlight the presence of a (positive or negative) relationship between some form of media use and a variable of interest. Correlations between pairs of variables may be misleading because those correlations might be explained by other variables. Linear regression on the other hand, implicitly assigns causal roles to the variables. Indeed, regressing, for example, academic performance on total hours of media is different from regressing total hours of media on academic performance and seems to suggest that total hours of media causes changes in academic performance. There are of course more advanced multivariate models which adequately treat measures as such (rather than implicitly assuming that some variables are measurement-free predictors, as is the case in linear regression) and explicitly define the directionality of the influence of the variables (most notably, structural equation models which include mediation and moderation models). However, there is currently no clear understanding of the relationships that might exist between the various constructs to justify a particular causal structure for such models.

This research field is currently characterized by both an ubiquity of correlations among variables and a paucity of evidence for specific causal relations among them. In this context, the method known as psychological network analysis [83, 84] seems particularly useful. This method is analogous to partial correlation analyses in that it attempts to evaluate the specific association between pairs of variables, albeit within the context of a network of variables. Using this method one may evaluate, for instance, whether there is a direct link between total media time and self-reported grades or whether the data is compatible with the hypothesis that total media time has an indirect effect on grades by reducing the amount or quality of sleep. Under certain assumptions, the presence of a direct association between two variables in a psychological network may be indicative of a causal relationship between them; however the directionality of the relationship remains unspecified. When there are many possible variables under investigation and no clear theoretical model, the associations highlighted in psychological networks might provide relevant starting points for future studies to investigate causality experimentally. While at this stage, this type of analysis is mostly exploratory, it offers a new perspective on previously reported effects and may constitute a promising avenue going forward.

In this study we collected data on three different self-reported measures of media use (total time on media, media multitasking and video game play) as well as a collection of measures that have been highlighted in past research; these measures cover attentional/behavioral control, mental health and school related variables. These questions were addressed in the 8–12 years age range, a particularly vulnerable time period of development for identity formation, socio-emotional development and, importantly for this work, further maturation of cognitive skills such as executive functions [52, 85]. In addition, it is during this age range that children transition to a more independent use of digital media, making it a specially interesting age range for researchers [52, 65]. This dataset includes a large set of variables for the same subjects, that is 156 eight-to-twelve year-old children. Although a larger sample size would always be welcomed, such multivariate dataset to qualify media usage in children remains rare in the literature.

We first probe the specificity of previously reported pairwise relationships between variables using rather traditional methods, such as partial correlation analysis, before using psychological network analysis. This two-step approach allows us to relate our results to past research while also providing new insights.

## Methods

### Ethics review and consent

The study was approved by the Ethics Review Commission of the University of Geneva. Parents provided written consent for their children to participate in this study.

### Participants

This study was conducted in a public primary school in the suburbs of Geneva, Switzerland. The school had expressed interest in participating in scientific studies. Children were recruited through teachers volunteering. These children were between 8 and 12 years old and were either in grade levels 5P to 8P (corresponding roughly to grades 3 to 6 in the United States [86]) or were part of a special needs class (we did not record or have access to any data further details characterizing children in this class). No inclusion/exclusion criteria were applied at data collection time. For the psychological network analyses reported below, we did not know what effect sizes to expect–instead we thrived to collect data from as many participants as we could, knowing that we would easily exceed the few tens of participants that are common in this type of study.

From the 226 children in the targeted classrooms, we obtained parental consent for 156 children (84 boys and 72 girls). Data from these 156 children are available on https://osf.io/aj2bc/. For the purposes of this study, we excluded from further analyses children from the special needs class (n = 16), children who did not complete the media questionnaire (n = 21). One additional child was excluded from further analyses because their reported number of daily hours on media (almost 35 hours) was much larger than for the remaining children (the second largest number was 17.5 hours)—note that it is possible to exceed 24 hours of media per day by consuming multiple media at the same time). This procedure ultimately led to an effective sample size of 118 children (57 girls, 61 boys, with a mean age of 10.38 years (SD = 1.16)). Note however that because of the multi-session nature of the study, some data are missing (e.g., children or their parents failed to complete one or more of the questionnaires or tests)—in the analyses below we report sample sizes for specific variables when relevant.

### Apparatus

We collected data via paper-and-pencil questionnaires completed by the parents and teachers of the children enrolled in this study as well as via cognitive tasks that were completed by the children in their classroom during school time. Below we list the surveys and cognitive tasks that were used.

We included a large amount of paper-and-pencil surveys to span a wide range of dimensions that might be relevant within the scope of this study. This selection included standard surveys from the literature but also custom-made questions that are more exploratory. The surveys were all administered in French translations of their original versions (our translations are available to readers by request). Children were asked to complete these questionnaires with their parents unless otherwise noted.

#### Questionnaires

In addition to a general demographic questionnaire—which asked children about their birthdate, gender, handedness, number of siblings, self-reported health state, the languages spoken at home, as well as yes/no questions about difficulties in vision, audition, learning and verbal comprehension and expression—the questionnaires included in this study cover broady speaking four categories: digital technology use; attentional problems; mental health and sleep; grades, motivation and beliefs. Below we present these questionnaires briefly; for more details, see the S1 File.

*Digital technology usage*. The **media multitasking inventory** is an adapted version of the media multitasking questionnaire [26]. We used three main measures from this questionnaire: the total number of hours of media content consumed per day, the media multitasking index (as defined in our study) and the total number of hours of video gaming per day.

The **video gameplay** questionnaire asks about which video games children play, on what device and how frequently (“often,” “sometimes,” “rarely”). Combined with the reported number of hours of video gaming from the media questionnaire, this survey provides an estimate of how much time is spent on each game category.

*Attentional problems*. The **Conners Teacher’s Rating Scale** [87] requires teachers to evaluate their children’s school behavior and leads to a score, where a higher value is interpreted as having overall more ADHD-like behavior (e.g., difficulty paying attention or impulsive behaviors).

The **Conners Parent’s Rating Scale** is similar but is filled out by the child’s parents.

We assessed **mind-wandering** or the frequency of task unrelated thoughts using the 4-item short Mind-Wandering Questionnaire (MWQ) [88].

**Mental health and sleep** The **K-6 distress scale** [89] evaluates non-specific psychological distress with items relating to anxiety and depression; a higher score reflects higher levels of emotional distress.

This **Strength and Difficulties questionnaire** [19] covers 5 dimensions of children’s behaviors, emotions, and relationships and provides a total score which reflects general difficulties, encompassing both emotional and behavioral problems.

We also included a custom-made **sleep** questionnaire from which we compute a score, with higher values indicating better sleep and less fatigue.

*Grades, grit and mindset*. The custom-made **grades** questionnaire asked children to self-report their grades (the question translates to “what do you think is your average general grade at school?”), and their grade satisfaction (“I have good grades at school” with responses on a four-point Likert scale going from yes to no).

The **grit** questionnaire measures perseverance and passion for long-term goals [55], with a higher score corresponding to greater perseverance.

Finally, the **Theory of Intelligence** (or mindset) questionnaire measures childrens’ beliefs about the potential of intelligence to improve [90]; a higher score indicates a “growth mindset” or a stronger belief that intelligence can be improved.

#### Cognitive tests

We report here 3 of the 5 cognitive tasks completed by the children in this study (one task was excluded because technical problems compromised the integrity of the data, and the other because it was part of an exploratory study that is unrelated to the present study). Each of these 3 tasks taps mostly attentional processes and gives rise to three main measures (for a total of 9 measures across the three tasks): a response speed index (how fast children perform), an inattention index (how often they fail to respond when they should have) and an impulsivity index (how often they respond when they shouldn’t have). These indices were then z-scored within tasks and averaged across tasks to provide an overall score on speed, inattention and impulsivity. Below we present these tests briefly; for more details, see the S1 File.

*D2 cancellation task*. The D2 task is a paper and pencil task designed to measure selective attention [91]. Participants were given a sheet of paper filled with symbols composed of the letters “d” or “p” with zero, one or two bars above and/or below the letter. Children were orally instructed to circle every symbol that comprises the letter “d” that is surrounded by exactly two bars (e.g., one above and one below; two above and none below).

*Sustained attention to response task (SART)*. In the SART task [92] a digit (1–9) appears for 250 ms on the screen center every 1.150ms and children are instructed to tap on the screen in response to any digit except the digit “3.”

*Bron lyon attention stability task (BLAST)*. In the BLAST task [93], children are first shown a single target letter (e.g., “A”) for 250ms, followed 500ms later by a 2x2 array of letters that did (e.g., “A, K, B, R”) or did not (e.g., “X, K, B, R”) contain the target letter. They were asked to report on each trial whether or not the target letter was present in the array.

### Procedure

The teachers distributed and collected the consent forms from the parents at the beginning of the school year. Parents could fill out the questionnaires at home with their child and bring them back to the school once they were completed. Teachers also filled out a questionnaire about each of their pupils whose parents consented to the study.

Given the large number of questionnaires and cognitive tests involved in this study, data collection was split into three sessions (January, March and May). In each session, different questionnaires and cognitive tests were completed.

The cognitive tests reported in this study were all conducted in a classroom setting where groups of 14 to 16 children were tested in a classroom setting under the supervision of at least two experimenters and a teacher. Each child was given their own tablet, pencil and paper on which the tests were implemented. They were seated at a table to complete the pencil and paper tests as well as computerized cognitive tests and were allowed to set the screen distance or position as they wished. This data collection procedure was motivated both by practical considerations and theoretical ones. At the practical level, it allowed for more efficient data collection. At the theoretical level, it allowed us to measure cognition in a real-life situation rather than an artificial lab setting. In doing so, our data collection is better aligned with our interest in understanding how media use affects everyday cognitive functioning. Each classroom test session lasted about 40 minutes. ## Code

Data processing, analyses and visualization were run in R version 3.6.0 (2019–04-26) [94], using the packages *tidyverse* [95], *lme4* [96], *lmerTest* [97] and *bootnet* [84]. For further details, see the S1 File. The code and data for this study are available on https://osf.io/aj2bc/.

## Results

Reliability and descriptive statistics are reported in the S1 File. Here we first briefly describe some key results about media usage among 8 to 12 year old children before evaluating specific relationships between different forms of media usages, attention, mental health and school related measures.

### Media consumption by age and gender

Total hours of media consumed per day increases with age (Spearman correlation r = 0.35, p < 0.001). At age 8, children consume on average 4 hours and 28 minutes of media per day; at age 12, that number increases to 8 hours and 14 minutes per day. For each additional year of age, total hours of media consumed increases by almost a full hour.

The total amount of media consumed does not differ among boys and girls (Wilcoxon rank sum test with continuity correction, W = 1669, p = 0.71). This result is confirmed by a linear mixed effects analysis on the hours spent per media by gender (F(1, 116) = 0.003, p = 0.957). Yet, this same analysis also shows that some media are consumed more than others (F(7, 812) = 16.1, p < 0.001) and differently by boys and girls (interaction effect, F(7, 812) = 5.6, p < 0.001). More specifically, boys spend more time on video games than girls (1.1±0.12 h/day versus 0.47±0.08 h/day; Wilcoxon rank sum test with continuity correction, W = 846, p < 0.001; gender differences for other media are smaller and would not resist multiple comparison correction).

Media multitasking also increases with age (Spearman correlation: r = 0.34, p < 0.001). At age 8, the media multitasking score—-which refers to the average number of additional media used while using a primary medium (a score of 0 meaning each medium is always consumed in isolation)—-is 0.66; at age 12, it increases to 1.61. For each additional year of age, the average number of additional media used simultaneously while using media increases by about 0.24.

Finally, there is no difference in media multitasking scores between boys and girls (Wilcoxon rank sum test with continuity correction, W = 1597, p = 0.613).

Within the age range studied, the number of hours spent on video games each day does not increase with age (Spearman correlation, r = 0.04, p = 0.695). Overall, boys spend more time on video games than girls do (mean±SEM: 1.1±0.12 versus 0.47±0.08 hours per day; Wilcoxon rank sum test with continuity correction, W = 846, p < 0.001). A linear mixed effects model on the hours of daily video gaming yields a significant interaction effect between gender and whether the games played were action-like or not (F(1, 186) = 12.6, p < 0.001): boys play more action-like video games than girls (W = 347, p < 0.001; 0.68±0.1 versus 0.13±0.05 hours per day) but there is no difference between them when considering time spent on other games (W = 1173.5, p = 0.535; 0.41±0.08 versus 0.5±0.1 hours per day).

### Attentional performance by age and gender

**Fig 1:**
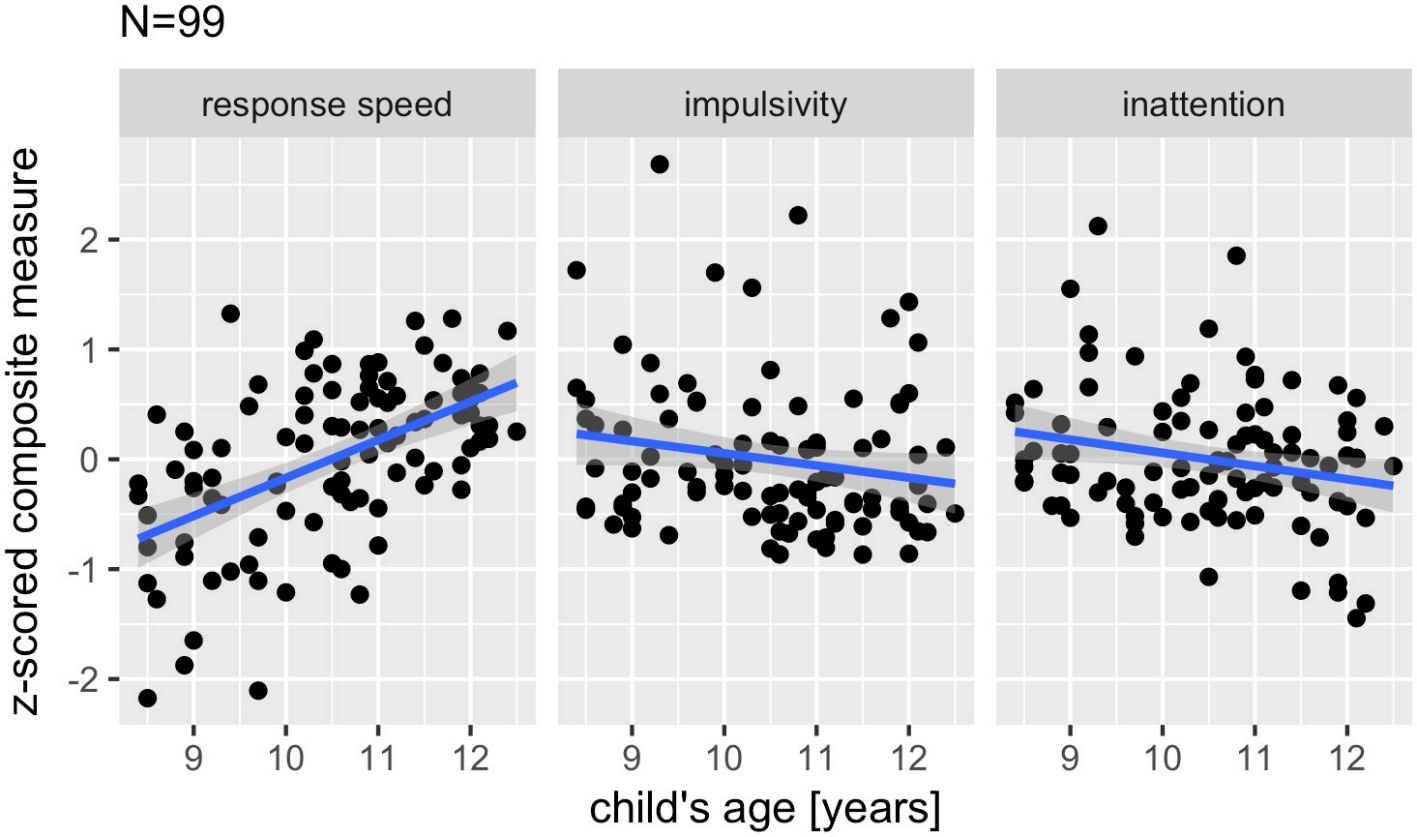
Cognitive performance as a function of children’s age.

As children get older, response speed increases (Spearman correlations: r = 0.52, p < 0.001), impulsivity decreases (i.e., tendency to make false alarms; r = -0.22, p = 0.029), and inattention decreases numerically although not in a statistically significant way according to the Spearman correlation test (i.e., miss rates; r = -0.15, p = 0.133; see Fig 1). To evaluate the effect of gender, in addition to age, we also ran linear regressions on the three cognitive indices (regressing separately the three cognitive indices on age, gender and their interaction). These analyses show a significant effect of age on speed (F(1,95) = 38.8, p < 0.001), on impulsivity (F(1,95) = 3.65, p = 0.059) and on inattention (F(1,95) = 5.14, p = 0.026). The effect of gender was significant on impulsivity (F(1,95) = 6.29, p = 0.014; with boys being more impulsive than girls) and on inattention (F(1,95) = 5.14, p = 0.026; with girls performing better) but not on response speed (F(1,95) = 3.22, p = 0.076). Finally, in none of the measures did we observe an interaction between age and gender (all p >= 0.208).

### Partial pairwise Spearman correlations

#### Total media time and media multitasking

As many of the variables are correlated—for example, response speed correlates with total hours of media consumed (r = 0.26, p = 0.008) but both response speed and total hours of media also correlate with age (r = 0.52, p < 0.001 and r = 0.35, p < 0.001)—it is not straightforward to interpret correlations between any pair of variables. We thus use partial correlations in an attempt to evaluate the specific relationship between two variables when controlling for age and other types of media use.

We computed the correlation between total hours of consumed media and each of our variables of interest while controlling for media multitasking and age and gender; we also did the reverse, i.e., compute the partial correlation between media multitasking and the variables of interest while controlling for total hours of media, age and gender. This procedure is justified by the fact that media multitasking and total hours of media are strongly correlated (r = 0.48, p < 0.001).

**Fig 2:**
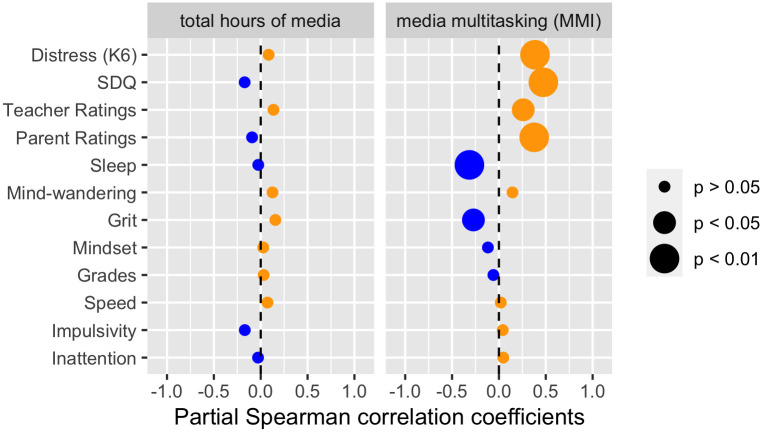
Partial Spearman correlation profiles of total hours of media (controlling for age, gender and MMI; left panel) and media multitasking (MMI; controlling for age, gender and total hours of media; right panel).

The partial correlation profiles corresponding to these two cases are shown in Fig 2. Clearly, these results show no correlation between total hours of media and any of the measures of interest when controlling for age, gender and media multitasking. However, when controlling for age, gender and total hours of media, we observe relationships between media multitasking scores and most self-reported measures. High levels of media multitasking are linked to higher levels of distress (K6), lower socioemotional functioning (SDQ), more behavioral and attentional problems as measured by both Conner’s Parents and Conner’s Teachers, as well as a reduced quality of sleep and lesser grit. No significant partial correlation is observed between media multitasking and mind-wandering, mindset, grades, or any of the cognitive performance measures.

#### Video gaming

Recall that the reported daily hours of video game play was taken from the media questionnaire and that there was a separate set of questions asking participants to report which games they played and at what frequency. This second questionnaire was used to determine if the games played by the children contained action-like mechanics or not (as well as other video game related data that are not reported here). From our data, we could compute the fraction of time spent on action-like video games versus other games. We then estimated the time spent on those two types of media by multiplying those fractions with the total daily hours of video game play. We excluded from this analysis participants who did not play at all (n = 21) or failed to report which specific games they played (n = 10).

We evaluated how playing video games relates to cognitive measures and found, in agreement with the literature, that video gaming correlates positively with response speed (r = 0.3, p = 0.006) but neither with impulsivity (r = -0.04, p = 0.746) nor inattention (r = 0.03, p = 0.793). The response speed effect appears mostly driven by time on action-like games (r = 0.22, p = 0.046) rather than other types of games (r = 0.13, p = 0.23; all other correlations are not statistically significant).

Next, we looked at the relationships between playing video games (overall and separating action and non-action video games) and our variables of interest, while controlling for age, gender, total hours of media and media multitasking score.

**Fig 3:**
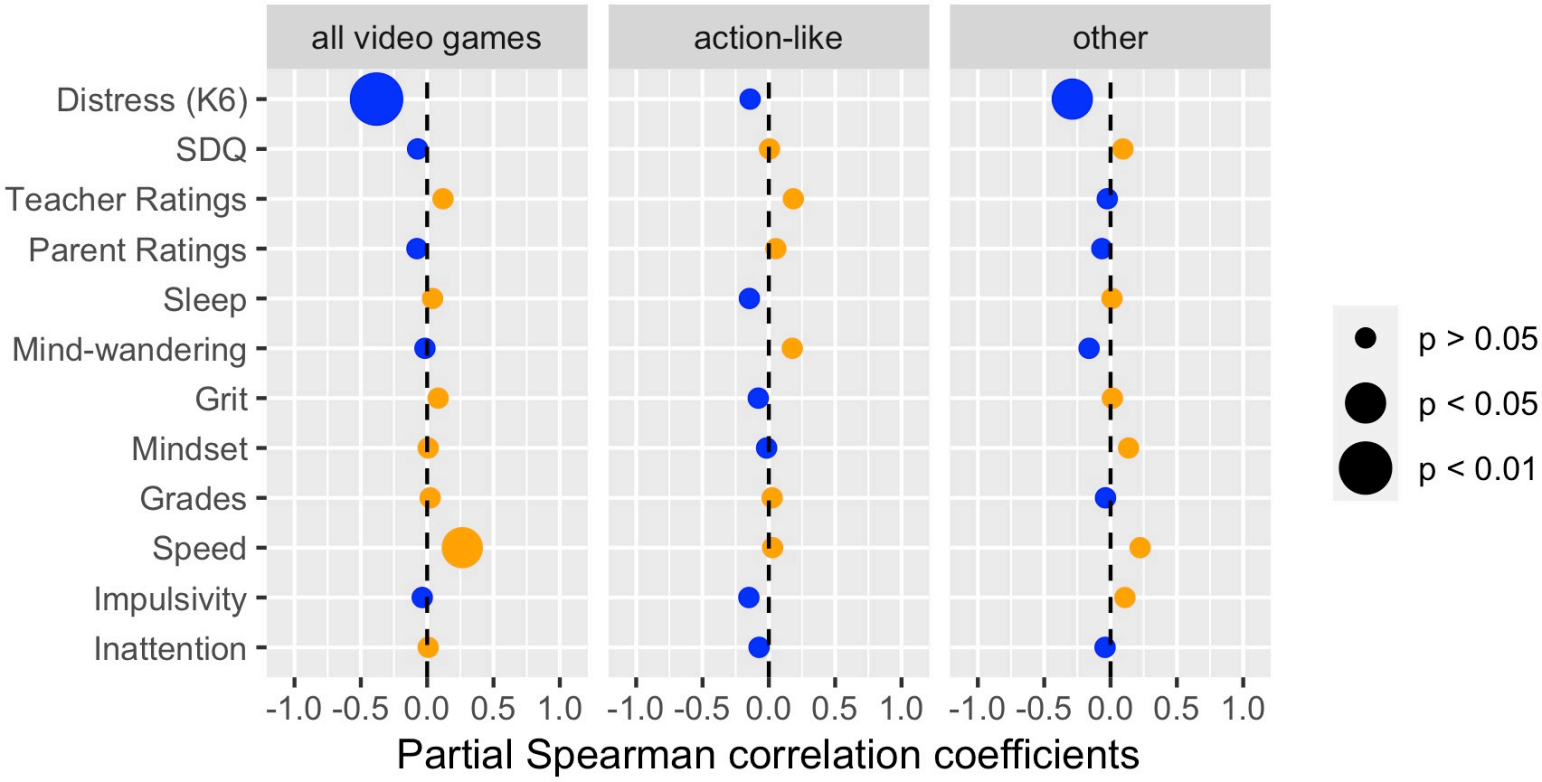
Partial Spearman correlation profiles of time spent on video games (controlling for age, gender and total hours of media and media multitasking). The three panels depict time spent on any kind of video games (left panel) or when considering separately time on action-like (middle panel) and non-action-like video games (right panel).

Overall, more time on video gaming is associated with faster response speed in the attentional control tasks (r = 0.26, p = 0.024, n = 77; see Fig 3) without, however, any concomitant increase in error rates that could have been indicative of an increased impulsivity or inattention (p > 0.756). The largest effect was observed on the K6 distress scale with more time on video game being associated with lower levels of distress (r = -0.38, p = 0.006, n = 55). These effects were only observed when collapsing all games together; with no clearly dissociable effects between action-like video games and other video games genres. No other reliable effects were observed.

### Psychological network

A generalization of the data analysis approach presented above consists in evaluating the partial correlations between any pair of variables while controlling for all the remaining variables; such a method is sometimes known as psychological network analysis. The resulting patterns of partial correlations may be represented as a network where nodes represent variables and edges the presence of a partial correlation between them [84]. Nodes connected by an edge therefore indicate a *direct* relationship between those variables, while indirect relationships are simply any non-direct pathway in the network. The advantage of this approach, compared to Structural Equation Modeling for example, is that it permits to simultaneously take into account a range of variables of interest without having to commit to a particular causal structure, which at this stage of the research remains largely unknown. It provides a picture of the complex relationships between various cognitive, demographic and life-style factors that might be a more accurate depiction of reality and less biased by a particular research agenda.

#### Description of the technique

Psychological Networks were estimated using the R package bootnet [84] and R [98]. The rationale is akin to estimating the partial correlation between each pair of variables while controlling for all remaining ones. However, as the number of possible partial correlations between each pair of variables increases rapidly with the number of variables, there is an increased chance of false positives (when not correcting for multiple testing) or a reduced probability to detect any effect at all (when controlling for multiple testing). An alternative approach, that circumvents these issues, estimates all the partial correlations at once and uses LASSO regularization and the Extended Bayesian Information Criterion to determine which model (with some of the partial correlations set to 0) best accounts for the observed data. There are no p-values associated with specific edges; rather, the estimated network as a whole, highlights the combination of edges that are reliable.

#### Variables entered into the analysis

Given our limited sample size and in order to limit spurious relationships, we included in this analysis only our primary variables for which we had the largest sample sizes. The eleven variables included in this analysis are the media multitasking score (N = 110), the total number of hours of media consumed each day (“Media Hours,” N = 118), the number of daily hours of video game play (“Gaming,” N = 118), child’s age and gender (as “Female,” N = 118), self-reported grades (“Grades,” N = 117), “Conner’s Teachers” (N = 92), “Sleep” score (N = 116) and the three composite scores from the attentional control task—i.e., “Speed,” “Inattention” and “Impulsivity” (N = 99).

### Network analysis

**Fig 4:**
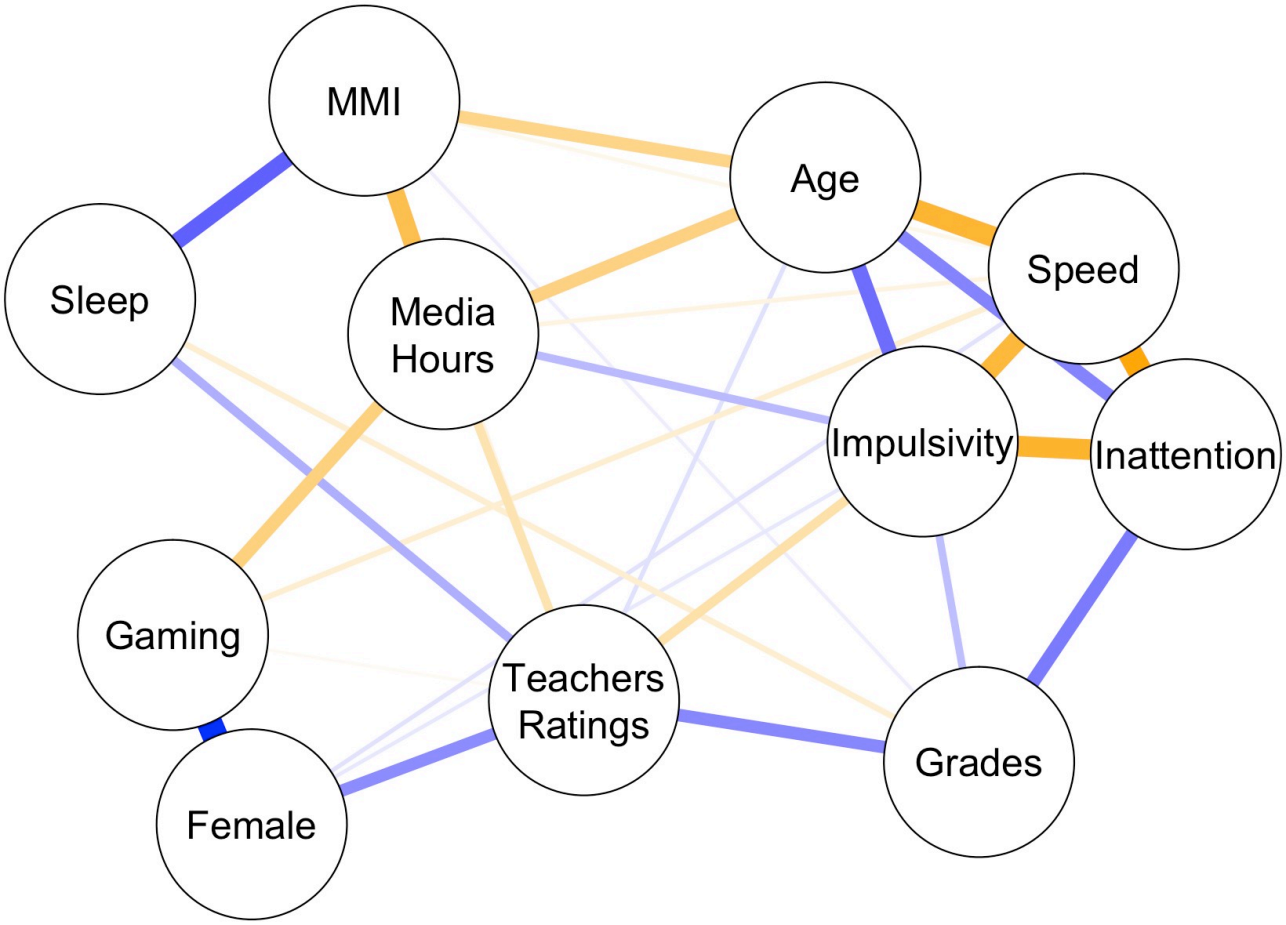
This concentration plot represents the network structure estimated on the 11 variables of interest. Nodes represent variables; edges represent the relationship between variables that cannot be explained by the remaining variables. The width and saturation of the edges reflect the strength of the relationship; positive associations are highlighted in orange and negative associations in blue (e.g., higher levels of media multitasking (MMI) are associated with being older, higher number of hours of media and worse sleep quality).

The concentration plot in Fig 4 highlights several noteworthy relationships. First, and as expected, age is strongly associated with the attentional control variables, being linked positively to speed and negatively to false-alarms (i.e., “Impulsivity”) and misses (i.e., “Inattention”). Thus, as expected, older children respond faster, and suffer less from impulsivity and inattention. Second, this analysis also confirms the well-known speed accuracy trade-off. Taking all variables into account, “Speed” is positively associated with “Impulsivity” and “Inattention” as faster participants tend to make more errors. Third, and also as expected, the three measures of technology use are positively related to each other, with time on media (i.e., “Media Hours”) being associated with both higher levels of media multitasking (“MMI”) and more time spent playing video games (“Gaming”). Interestingly, no direct relationship between “Gaming” and “MMI” is observed. Furthermore, there is a positive association with age for both “Media Hours” and “MMI”—indicating greater technology consumption as children get older.

Of greater practical interest are the predictors of “Grades.” Both lower “Inattention” and lower “Impulsivity” levels are associated with better grades—“Speed” however is not directly related to grades. Furthermore, grades relate directly to “Teacher Ratings” with, as expected, higher ratings (i.e., higher levels of behavioral issues and attentional deficits) being associated with lower grades. “Teacher Ratings” are associated with “Impulsivity” as measured in the attentional control tasks, which attest to the consistency and validity of these measures. “Teacher Ratings” also relate to the child’s gender—-with girls receiving better ratings than boys-—and to sleep habits-—with poor self-reported sleep satisfaction being associated with worse teacher rated attentional/behavioral problems (i.e., higher “Teacher Ratings” scores).

Technology use is found to have little to no *direct* relationship with grades, except for MMI—higher levels of MMI are weakly associated with lower grades. Most relationships between media uses and grades appear instead to be indirect: higher levels of MMI are associated with poor sleep and with more hours of media consumed; worse sleep and more hours of media consumed are associated with worse teacher rated attentional/behavioral problems, which in turn are associated with lower grades. Total hours of media is only indirectly related to grades via worse teacher ratings. Finally, there are no clear links between gaming and either grades, teacher ratings, or sleep. Gaming is only associated with increased response speed, albeit weakly.

## Discussion

There are growing concerns about the potential of digital media to negatively impact everyday life functioning, in particular during childhood. These concerns call for empirical data on media use in children that is both granular (i.e., considering separate forms of media use), comprehensive (i.e., considering simultaneously a wide range of possible outcome variables) and which adequately handles the fact that many of these variables correlate with each other. Here we investigated three main aspects of media consumption behavior—total media hours, media multitasking and video gaming—among a population of 8 to 12-year old children. The present work addresses the relationships between these three distinct forms of media consumption and attentional and behavioral control (measured through both cognitive tests and questionnaires), mental health and sleep, grades, grit and mindset.

Our results confirm the well-established observation that as children age, they consume more media. In this representative sample of Swiss children, the amount of media content consumed each day increased steadily by one additional daily hour per year of age. From ages 8 to 12, the daily hours of media consumed increased from about 5 to 9 hours. This is in line with many reports in the literature [1], including Pea et al. [52] who used a similar (albeit online) survey on a sample of 3,461 8 to 12 year old girls and reported an average of 6.9 hours of daily total media use. This was concomitant with an increase in the number of media used at the same time by 1.4 additional media. In our work, the average number of additional media that children use when using more than one medium at the same time increased from a value of 0.66 at age 8 to a value of 1.61 at age 12. Notably, girls and boys did not differ in terms of total media time or amount of media multitasking—-unlike other studies reporting that girls media multitask more than boys [48, 99]. Girls and boys did however differ in the types of media consumed, with boys reporting larger amounts of video game play, especially those containing action-like mechanics. The extent to which these differences in media consumption foster gender differences in cognitive skills remains an interesting open question [100, 101].

### Total media time and its limitations as a metric

We argue, as have many before us [6, e.g., 9–11], that total time on media is not a sufficient metric. Total time on media correlates strongly with measures of more specific forms of media use, each of which having their unique, positive or negative impact. This is apparent in the psychological network analysis where total time on media is among the most connected nodes. Yet, this analysis also highlights the distinctness of media multitasking and video gaming, each of them in separate clusters. The shared variance captured by total time on media appears determinant to account for poor attentional behavior. Children who spend more time on media are more frequently reported by their teachers to manifest ADHD-like behavior. Conceptually, this relationship is in line with past research [16, 17, 82]. Yet, partial correlation analyses reveal that media multitasking might be driving this effect. We observe no significant relationships between total time on media and any of our outcome variables when controlling for media multitasking, age, and gender. Although these results could appear to contradict those published in the past [15, 82, 102], these past studies used total media time without controlling for other types of media consumption. In contrast to total media time, media multitasking is associated with more frequent ADHD-like behavior as rated by their teachers, when controlling for total media time, gender, and age.

### Media multitasking versus video game play

The partial pairwise correlations highlight large and significant partial correlations linking media multitasking with numerous adverse, self-reported measures: higher levels of media multitasking were associated with higher levels of psychological distress (K6), lower levels of socioemotional functioning (SDQ), worse behavior and attention ratings by both teachers and parents, worse sleep and lower levels of grit. These results are in line with past research reporting an association between media multitasking and increased depression and anxiety among young adults after controlling for total time on media and various personality traits [50], or media multitasking and worse socioemotional outcomes and worse sleep among 8- to 12- year old girls [52]. The psychological network analysis further supports the negative link between media multitasking and worse sleep. Sleep is important because it is known to affect many aspects of our lives, including attentional/behavioral control, mental health and school related variables [for reviews see, [103, 104]. In agreement with that literature, in our study, children who report worse sleep both have worse grades and receive less favorable attention and behavior ratings from their teachers. A relationship between higher levels of media multitasking and worse sleep has already been reported several times, both in children [52] and adolescents [51, 53], with some researchers suggesting that sleep is more strongly associated with media multitasking than with total time on media [53]—this is also the case here, as we observe no relationship between total time on media and sleep.

Contrary to the analysis on media multitasking, both partial correlation analyses on hours of video gaming (controlling for age, gender, total time of media consumed and media multitasking index) and the psychological network analyses revealed no significant adverse associations. More specifically, we observed no significant partial correlation involving video gaming and socioemotional functioning, attention and behavioral issues as rated by teachers and parents, sleep, mind-wandering, grit, growth mindset, grades and either impulsivity and inattention in cognitive tests. Rather, we found positive relationships between time spent playing video games and both faster response speed in our attentional control tests, and reduced levels of psychological distress; indicating that playing video games might have a positive impact on specific measures of cognitive control and mental health. The psychological network analysis (which included only a subset of the variables listed above) depicted a similar pattern of results, showing that more time spent on video games is associated with increased response speed in attention tests, in addition to being a male and spending more time on media overall.

When considering video game play in general, our results are partially in agreement with the literature which so far has yielded mixed results. Pujol et al. [69] for instance, tested over two thousand 7- to 11- year old children using a somewhat similar protocol to ours: children completed cognitive tests (of attention and working memory) and their parents and teachers filled out questionnaires about children’s video gaming habits, their socioemotional functioning (using the Strength and Difficulties Questionnaire), their sleep and overall school achievement. In agreement with our results, their study shows that playing video games was linked with increased response speed without however affecting overall performance on the cognitive tests. Contrary to our results however, they observed a link between video gaming and sleep (more time on video games was linked to sleeping fewer hours). It remains unclear in the Pujol dataset, whether this relationship may be related to video gaming per se or to other associated variables like media multitasking.

In our study we found no direct relationship between time spent on video games and grades. Both the evidence and the opinions in the literature on how video gaming relates to academic performance are somewhat mixed. Some data on children and adolescents is compatible with video gaming being associated with greater school achievement [69, 71], while other suggests either no relationship or a small/moderate negative relationship for those children who play video games before going to school [7, 73, 75]. These mixed results are paralleled by different opinions on what relationship to expect. On the one hand, researchers have argued that some kinds of video games improve cognitive abilities which are thought to be crucial for academic performance [105]; on the other, researchers expect video gaming to negatively impact academic performance by either taking away time from other activities, impairing sleep or the ability to concentrate in a slow paced school environment [e.g., 75, 106]. The present data do not support the view that video gaming impairs academic achievement as we find no direct relationship between video gaming and grades as well as no direct relationship between video gaming and sleep (we do however see the well-known link between better sleep quality and better grades). Gaming might affect educational performance by improving cognition, albeit indirectly, given the link between video gaming and cognition (increased speed) and the link between cognition and grades (lower impulsivity and inattention are linked to better grades).

While our study does not resolve the many inconsistencies in the field of media and their impact on cognitive functioning, mental health, and school related variables, it clearly highlights a few major points. First, it is absolutely necessary to take into account not only total time on media, but also other, more specific measures of media consumption. Here we considered media multitasking and video game playing (in addition to total media time); it seems highly valuable for future studies to include social media, internet browsing or TV/video watching to cite a few. From this point of view, media multitasking questionnaires could be further exploited to document these different forms of media consumption. Second, in the case of video games, the specific types of games that are played (e.g., action-like versus non-action), how and when they are played (e.g., before or after school; in the morning versus evening) appear to be important factors to consider. As different video games have been shown to differently affect cognition, considering their impact separately may help explain some of the discrepancies in the literature [4, 68]. Third, while most empirical research focused on pairwise relationships (e.g., between playing video games and grades), researchers do in fact have implicit or explicit hypotheses of how specifically these variables relate (e.g., gaming improves educational attainment by improving attentional control)—the psychological network analysis presented in this study emerges as a powerful complementary tool to evaluate the plausibility of these hypotheses along with pairwise correlation.

### Psychological network analysis as a promising tool to study media effects on humans

The psychological network analysis applied on this data set confirmed several well-known results (unrelated to media use), further underlining the value of the approach. For instance, age was associated with increased attentional control abilities [increased response speed, reduced impulsivity and reduced inattention; [107]]. Factoring out age, increased response speed was associated with greater impulsivity and greater inattention (as measured by more frequent false alarms and misses, respectively); this pattern of results reflects the well-known speed-accuracy tradeoff phenomenon. We also observed the expected relationships between academic grades and children’s behavioral and attentional problems as rated by their teachers. Inattention, impulsivity, worse teacher rated attentional/behavioral problems and reduced sleep quality were unsurprisingly also directly associated with lower grades [108]. Worse teacher rated attentional/behavioral problems were in turn associated with inattention, lower sleep quality and being male [e.g., 109]. Psychological network analysis therefore appears to be a very powerful tool to shed light on the relationships between multiple variables. It seems particularly well-suited to study the relationships between media consumption, attentional/behavioral control, mental health and academic achievement because many of these variables correlate with each other and their causal relationships remain largely unresolved. Applying psychological network analyses on a larger set of variables might provide a means to untangle some of the past results.

### Limitations of the present study

We recognize this study has several important limitations. First, all the data reported here are correlational and as such provide no unequivocal indication about the causal relationships between any of the variables of interest. In particular, our study highlights that different types of media use are far from independent, calling for care when separating out direct relations. For example, media multitasking has been repeatedly associated with a greater tendency to mind-wander [37, but see 38] and we did observe such a relationship as well in simple pairwise correlations (pairwise Spearman correlations: r = 0.31, p = 0.012). Yet, we also observed a correlation between mind-wandering and total hours of consumed media (r = 0.31, p = 0.009) which itself correlates with media multitasking (for completeness, note that hours of video gaming does not correlate with mind-wandering; r = 0.16, p = 0.186). Such a pattern of results highlights the fact that it may be problematic to attribute the variations in mind-wandering to media multitasking rather than to total hours of consumed media. Causal studies would certainly be highly valuable but may not be ethical given some of the negative impacts reported here and in the broader literature.

Second, given the number of tests and surveys used in the present study, it would have been desirable to include data from a larger population. For instance, while we have a nested structure for analysis (individual children are grouped in classes), we do not have the necessary size to perform such an analysis robustly. Third, while research has repeatedly indicated that the effects of video games on cognition depend largely on the video game genre [59–61, 63, 64], we did not have sample sizes large enough to substantiate any claims regarding genre-specific effects. The modest or absent effects observed in the present study might turn out very differently if video gaming activity is considered with greater minutiae (e.g., action, social, puzzle). Fourth, although this study included numerous measures it is likely that important measures were missed and deserve to be investigated in future studies. For instance, social media use is not expected to have the same attentional/behavioral or mental health impact as playing video games [110–112]. Finally, many of the measures collected in this study are self-reports and as such are subject to biases. It is well known, for instance, that self-reported media use questionnaires do not fully reflect real usage [e.g., 113–115]. Objective measures of media consumption, such as event-sampling or usage monitoring, would be preferable [116].

### Future perspectives

Understanding how digital technologies impact our lives is extremely challenging because of the richness and ever changing landscape of digital media. Indeed, there are many ways the same technology may be used, multitude of facets in our daily lives that technologies may impact (e.g., mental health, cognition, well-being to name a few) and numerous routes through which these variables may interact with each other. Getting a better grasp of the effects of technology use will require new approaches including the development of benchmark tools both in terms of use measurement and impact, and the collection of larger datasets most likely within multi-laboratories initiatives. An important step towards that goal will involve clarifying concepts and building frameworks to characterize both technology use as well as psychological constructs of interest–an exemplary case in this context is the work conducted by Meier and Reineck [9] who proposed a hierarchy of six levels of analysis to structure research in this field: device (e.g., tablet versus laptop), type of application (e.g., email versus video), branded application (e.g., Facebook versus Instagram), feature (e.g., messenger versus chat), interaction (e.g., synchronous versus asynchronous) and message (e.g., text versus voice). It is encouraging to see that coming from a rather different, cognitive perspective, [11] have converged on a rather similar and complementary set of analysis levels, highlighting in addition the importance of content (e.g., social simulation game versus war-based games), context of use (e.g., single media versus multitasking; alone or in a social context), and user characteristics (e.g., children versus adults; typically developing versus with learning disabilities). Such frameworks and taxonomies will be useful for building unifying theories, making sense of the puzzle pieces collected so far but also to guide research in a more systematic way.

### Conclusion

This study shows that different aspects of media consumption have different relationships with attentional/behavioral outcomes, mental health and school relevant variables, and thus highlights the importance of using more granular assessments than just total media time.

It is not uncommon to read that time in front of screens should be limited. The present paper indicates that such aggregate measures of media consumption are not sufficient and documents that the type of media used as well as how they are consumed both matter. In this study, we are able to highlight such differences through multiple related, but distinct, media use measures, and by using partial correlations and psychological networks. These analyses reveal that media multitasking more than video gaming and total time on media was associated with adverse psychological outcomes and that media multitasking should therefore be considered more intensively in future studies.

Finally, the complexity of measuring media consumption calls for a paradigm shift that integrates real-life usage sampling, event sampling along with self-reports. Given the rapidly changing landscape of digital media and the complexity of the topic it would seem beneficial for the field to coordinate multi-lab studies and to systematically share data and methods on best practice to representatively and usefully sample media consumption.

## Supporting information

S1 File Supplementary materials. This file contains additional information about the methods and dataset as well as supplementary analyses.
(PDF)

## References

[1] Rideout V. The Common Sense Census: Media Use by Tweens and Teens [Internet]. San Francisco: Common Sense Media; 2015 p. 104. Available: https://www.commonsensemedia.org/sites/default/files/uploads/research/census_researchreport.pdf

[2] Rideout VJ, Foehr UG, Roberts DF. Generation M 2: Media in the Lives of 8-to 18-Year-Olds. Henry J Kaiser Family Foundation. ERIC; 2010

[3] Bodson L. Regards sur les activités quotidiennes des jeunes résidents. Luxembourg: Institut national de la statistique et des études économiques (STATEC); 2017.

[4] Cardoso-Leite P, Ansarinia M, Schmück E, Bavelier D. Training cognition with video games. In: Cohen Kadosh K, editor. The Oxford Handbook of Developmental Cognitive Neuroscience. Oxford University Press; 2021. 10.1093/oxfordhb/9780198827474.013.38

[5] Bavelier D, Green CS. Enhancing Attentional Control: Lessons from Action Video Games. Neuron. 2019 doi: 10.1016/j.neuron.2019.09.031 31600511

[6] Granic I, Morita H, Scholten H. Beyond Screen Time: Identity Development in the Digital Age. Psychological Inquiry. Routledge; 2020;31: 195–223. doi: 10.1080/1047840X.2020.1820214

[7] Adelantado-Renau M, Moliner-Urdiales D, Cavero-Redondo I, Beltran-Valls MR, Martínez-Vizcaíno V, Álvarez-Bueno C. Association Between Screen Media Use and Academic Performance Among Children and Adolescents: A Systematic Review and Meta-analysis. JAMA Pediatr. 2019;173: 1058. doi: 10.1001/jamapediatrics.2019.3176 31545344 PMC6764013

[8] Beyens I. The effect of social media on well-being differs from adolescent to adolescent. Scientific Reports. 2020; 11. 10.1038/s41598-020-67727-7 PMC7329840 32612108

[9] Meier A, Reinecke L. Computer-Mediated Communication, Social Media, and Mental Health: A Conceptual and Empirical Meta-Review. Communication Research. 2020; 009365022095822. doi: 10.1177/0093650220958224

[10] Orben A, Weinstein N, Przybylski AK. Only Holistic and Iterative Change Will Fix Digital Technology Research. Psychological Inquiry. Routledge; 2020;31: 235–241. doi: 10.1080/1047840X.2020.1820221

[11] Bediou B, Rich M, Bavelier D. Digital media and cognitive development. OECD; 2020; https://doi-org.proxy.bnl.lu/10.1787/3b071e13-en

[12] Twenge JM, Campbell WK. Associations between screen time and lower psychological well-being among children and adolescents: Evidence from a population-based study. Preventive Medicine Reports. 2018;12: 271–283. doi: 10.1016/j.pmedr.2018.10.003 30406005 PMC6214874

[13] Allen MS, Vella SA. Screen-based sedentary behaviour and psychosocial well-being in childhood: Cross-sectional and longitudinal associations. Mental Health and Physical Activity. 2015;9: 41–47. doi: 10.1016/j.mhpa.2015.10.002

[14] Beyens I, Valkenburg PM, Piotrowski JT. Screen media use and ADHD-related behaviors: Four decades of research. Proceedings of the National Academy of Sciences. 2018;115: 9875–9881. doi: 10.1073/pnas.1611611114 30275318 PMC6176582

[15] Zhao J, Zhang Y, Jiang F, Ip P, Ho FKW, Zhang Y, et al. Excessive Screen Time and Psychosocial Well-Being: The Mediating Role of Body Mass Index, Sleep Duration, and Parent-Child Interaction. The Journal of Pediatrics. 2018;202: 157–162.e1. doi: 10.1016/j.jpeds.2018.06.029 30100232

[16] Ra CK, Cho J, Stone MD, De La Cerda J, Goldenson NI, Moroney E, et al. Association of Digital Media Use With Subsequent Symptoms of Attention-Deficit/Hyperactivity Disorder Among Adolescents. JAMA. 2018;320: 255. doi: 10.1001/jama.2018.8931 30027248 PMC6553065

[17] Nikkelen SWC, Valkenburg PM, Huizinga M, Bushman BJ. Media use and ADHD-related behaviors in children and adolescents: A meta-analysis. Developmental Psychology. 2014;50: 2228–2241. doi: 10.1037/a0037318 24999762

[18] Limtrakul N, Louthrenoo O, Narkpongphun A, Boonchooduang N, Chonchaiya W. Media use and psychosocial adjustment in children and adolescents. J Paediatr Child Health. 2018;54: 296–301. doi: 10.1111/jpc.13725 28948669

[19] Goodman R. The Strengths and Difficulties Questionnaire: A research note. J Child Psychol Psychiatry. 1997;38: 581–586. doi: 10.1111/j.1469-7610.1997.tb01545.x 9255702

[20] Domoff SE, Harrison K, Gearhardt AN, Gentile DA, Lumeng JC, Miller AL. Development and Validation of the Problematic Media Use Measure: A Parent Report Measure of Screen Media “Addiction” in Children. Psychol Pop Media Cult. 2019;8: 2–11. doi: 10.1037/ppm0000163 30873299 PMC6411079

[21] Orben A, Przybylski AK. The association between adolescent well-being and digital technology use. Nature Human Behaviour. 2019 doi: 10.1038/s41562-018-0506-1 30944443

[22] Aypay A. Information and Communication Technology (ICT) Usage and Achievement of Turkish Students in Pisa 2006. Turkish Online Journal of Educational Technology—TOJET. 2010;9: 116–124. Available: https://eric.ed.gov/?id=EJ898009

[23] Bulut O, Cutumisu M. When Technology Does Not Add Up: ICT Use Negatively Predicts Mathematics and Science Achievement for Finnish and Turkish Students in PISA 2012. Association for the Advancement of Computing in Education (AACE); 2017. pp. 935–945. Available: https://www.learntechlib.org/primary/p/178407/

[24] Rideout VJ, Vandewater EA, Wartella EA. Zero to six: Electronic media in the lives of infants, toddlers and preschoolers. ERIC; 2003 10.1542/peds.2006-1804 17473074

[25] Roberts D, Foehr U, Rideout V. Generation M: Media in the Lives of 8-18 Year-Olds, Kaiser Family Foundation. March http://www.kff.org/entmedia/entmedia030905nr.cfm. 2005

[26] Ophir E, Nass C, Wagner AD. Cognitive control in media multitaskers. PNAS. 2009;106: 15583–7. doi: 10.1073/pnas.0903620106 19706386 PMC2747164

[27] Wiradhany W, Nieuwenstein MR. Cognitive control in media multitaskers: Two replication studies and a meta-Analysis. Attention, Perception, and Psychophysics. Attention, Perception, & Psychophysics; 2017 doi: 10.3758/s13414-017-1408-4 28840547 PMC5662702

[28] Uncapher MR, Lin L, Rosen LD, Kirkorian HL, Baron NS, Bailey K, et al. Media Multitasking and Cognitive, Psychological, Neural, and Learning Differences. Pediatrics. 2017;140: S62–S66. doi: 10.1542/peds.2016-1758D 29093034 PMC5658797

[29] Uncapher MR, Wagner AD. Minds and brains of media multitaskers: Current findings and future directions. Proceedings of the National Academy of Sciences. 2018;115: 9889–9896. doi: 10.1073/pnas.1611612115 30275312 PMC6176627

[30] Cheever NA, Peviani K, Rosen LD. Media Multitasking and Mental Health. In: Moreno MA, Radovic A, editors. Technology and Adolescent Mental Health. Cham: Springer International Publishing; 2018. pp. 101–112. 10.1007/978-3-319-69638-6_8

[31] Magen H. The relations between executive functions, media multitasking and polychronicity. Computers in Human Behavior. 2017;67: 1–9. doi: 10.1016/j.chb.2016.10.011

[32] Uncapher MR, Thieu MK., Wagner AD. Media multitasking and memory: Differences in working memory and long-term memory. Psychonomic Bulletin & Review. 2016;23: 483–490. doi: 10.3758/s13423-015-0907-3 26223469 PMC4733435

[33] Baumgartner SE, Sumter SR. Dealing with media distractions: An observational study of computer-based multitasking among children and adults in the Netherlands. Journal of Children and Media. 2017;11: 295–313. doi: 10.1080/17482798.2017.1304971

[34] Minear M, Brasher F, McCurdy M, Lewis J, Younggren A. Working memory, fluid intelligence, and impulsiveness in heavy media multitaskers. Psychonomic bulletin & review. 2013;20: 1274–81. doi: 10.3758/s13423-013-0456-6 23722949

[35] Sanbonmatsu DM, Strayer DL, Medeiros-Ward N, Watson JM. Who Multi-Tasks and Why? Multi-Tasking Ability, Perceived Multi-Tasking Ability, Impulsivity, and Sensation Seeking. Chambers C, editor. PLoS ONE. 2013;8: e54402. doi: 10.1371/journal.pone.0054402 23372720 PMC3553130

[36] Shin M, Webb A, Kemps E. Media multitasking, impulsivity and dual task ability. Computers in Human Behavior. 2019;92: 160–168. doi: 10.1016/j.chb.2018.11.018

[37] Ralph BCW, Thomson DR, Cheyne JA, Smilek D. Media multitasking and failures of attention in everyday life. Psychological Research. 2014;78: 661–669. doi: 10.1007/s00426-013-0523-7 24178629

[38] Wiradhany W, van Vugt MK, Nieuwenstein MR. Media multitasking, mind-wandering, and distractibility: A large-scale study. Atten Percept Psychophys. 2019. doi: 10.3758/s13414-019-01842-0 PMC7303060 31392594

[39] Kobayashi K, Oishi N, Yoshimura S, Ueno T, Miyagi T, Murai T, et al. Relationship between media multitasking and functional connectivity in the dorsal attention network. Scientific Reports. Nature Publishing Group; 2020;10: 17992. doi: 10.1038/s41598-020-75091-9 33093496 PMC7582949

[40] Madore KP, Khazenzon AM, Backes CW, Jiang J, Uncapher MR, Norcia AM, et al. Memory failure predicted by attention lapsing and media multitasking. Nature. Nature Publishing Group; 2020;587: 87–91. doi: 10.1038/s41586-020-2870-z 33116309 PMC7644608

[41] Alzahabi R, Becker MW. The association between media multitasking, task-switching, and dual-task performance. Journal of Experimental Psychology: Human Perception and Performance. 2013;39: 1485–1495. 23398256 10.1037/a0031208

[42] Elbe P, Sörman DE, Mellqvist E, Brändström J, Ljungberg JK. Predicting attention shifting abilities from self-reported media multitasking. Psychon Bull Rev. 2019;26: 1257–1265. doi: 10.3758/s13423-018-01566-6 31030392 PMC6710456

[43] Seddon AL, Law AS, Adams A-M, Simmons FR. Exploring the relationship between executive functions and self-reported media-multitasking in young adults. Journal of Cognitive Psychology. Routledge; 2018;30: 728–742. doi: 10.1080/20445911.2018.1525387

[44] Cardoso-Leite P, Kludt R, Vignola G, Ma WJ, Green CS, Bavelier D. Technology consumption and cognitive control: Contrasting action video game experience with media multitasking. Attention, Perception, & Psychophysics. 2016;78: 218–241. Available: http://link.springer.com/article/10.3758/s13414-015-0988-0 26474982 10.3758/s13414-015-0988-0 PMC4834063

[45] Shin M, Linke A, Kemps E. Moderate amounts of media multitasking are associated with optimal task performance and minimal mind wandering. Computers in Human Behavior. 2020;111: 106422. doi: 10.1016/j.chb.2020.106422

[46] Baumgartner SE, Weeda WD, van der Heijden LL, Huizinga M. The Relationship Between Media Multitasking and Executive Function in Early Adolescents: The Journal of Early Adolescence. 2014 doi: 10.1177/0272431614523133

[47] Rogobete DA, Ionescu T, Miclea M. The Relationship Between Media Multitasking Behavior and Executive Function in Adolescence: A Replication Study. The Journal of Early Adolescence. SAGE Publications Inc; 2020; 0272431620950478. doi: 10.1177/0272431620950478

[48] Baumgartner SE, van der Schuur WA, Lemmens JS, te Poel F. The Relationship Between Media Multitasking and Attention Problems in Adolescents: Results of Two Longitudinal Studies. Human Communication Research. 2018;44: 3–30. doi: 10.1093/hcre.12111

[49] Srisinghasongkram P, Trairatvorakul P, Maes M, Chonchaiya W. Effect of early screen media multitasking on behavioural problems in school-age children. Eur Child Adolesc Psychiatry. 2020 32856131 10.1007/s00787-020-01623-3

[50] Becker MW, Alzahabi R, Hopwood CJ. Media Multitasking Is Associated with Symptoms of Depression and Social Anxiety. Cyberpsychology, Behavior, and Social Networking. 2013;16: 132–135. doi: 10.1089/cyber.2012.0291 23126438

[51] Calamaro CJ, Mason TBA, Ratcliffe SJ. Adolescents Living the 24/7 Lifestyle: Effects of Caffeine and Technology on Sleep Duration and Daytime Functioning. PEDIATRICS. 2009;123: e1005–e1010. doi: 10.1542/peds.2008-3641 19482732

[52] Pea R, Nass C, Meheula L, Rance M, Kumar A, Bamford H, et al. Media use, face-to-face communication, media multitasking, and social well-being among 8- to 12-year-old girls. Developmental Psychology. 2012;48: 327–336. doi: 10.1037/a0027030 22268607

[53] van der Schuur WA, Baumgartner SE, Sumter SR, Valkenburg PM. Media multitasking and sleep problems: A longitudinal study among adolescents. Computers in Human Behavior. 2018;81: 316–324. doi: 10.1016/j.chb.2017.12.024

[54] Loh KK, Tan BZH, Lim SWH. Media multitasking predicts video-recorded lecture learning performance through mind wandering tendencies. Computers in Human Behavior. 2016;63: 943–947. doi: 10.1016/j.chb.2016.06.030

[55] Duckworth AL, Peterson C, Matthews MD, Kelly DR. Grit: Perseverance and passion for long-term goals. Journal of Personality and Social Psychology. 2007;92: 1087–1101. doi: 10.1037/0022-3514.92.6.1087 17547490

[56] Cain MS, Leonard JA, Gabrieli JDE, Finn AS. Media multitasking in adolescence. Psychonomic Bulletin & Review. 2016;23: 1932–1941. doi: 10.3758/s13423-016-1036-3 27188785

[57] Martín-Perpiñá M de las M, Viñas i Poch F, Malo Cerrato S. Media multitasking impact in homework, executive function and academic performance in Spanish adolescents. Martin-Perpiñá, Mercedes Viñas i Poch, Ferran Malo Cerrato, Sara 2019 Media multitasking impact in homework, executive function and academic performance in Spanish adolescents Psicothema 31 1 81 87. Colegio Oficial de Psicólogos del Principado de Asturias; 2019 10.7334/psicothema2018.178 30664415

[58] Luo J, Yeung P, Li H. The relationship among media multitasking, academic performance and self-esteem in Chinese adolescents: The cross-lagged panel and mediation analyses. Children and Youth Services Review. 2020;117: 105308. doi: 10.1016/j.childyouth.2020.105308

[59] Powers KL, Brooks PJ. Evaluating the Specificity of Effects of Video Game Training. In: Blumberg FC, editor. Learning by Playing. Oxford University Press; 2014. pp. 302–330. 10.1093/acprof:osobl/9780199896646.003.0021

[60] Powers KL, Brooks PJ, Aldrich NJ, Palladino MA, Alfieri L. Effects of video-game play on information processing: A meta-analytic investigation. Psychon Bull Rev. 2013;20: 1055–1079. doi: 10.3758/s13423-013-0418-z 23519430

[61] Wang P, Liu H-H, Zhu X-T, Meng T, Li H-J, Zuo X-N. Action Video Game Training for Healthy Adults: A Meta-Analytic Study. Front Psychol. 2016;7. doi: 10.3389/fpsyg.2016.00907 27378996 PMC4911405

[62] Toril P, Reales JM, Ballesteros S. Video game training enhances cognition of older adults: A meta-analytic study. Psychology and Aging. 2014;29: 706–716. doi: 10.1037/a0037507 25244488

[63] Bediou B, Adams DM, Mayer RE, Tipton E, Green CS, Bavelier D. Meta-analysis of action video game impact on perceptual, attentional, and cognitive skills. Psychological Bulletin. 2018;144: 77–110. doi: 10.1037/bul0000130 29172564

[64] Sala G, Tatlidil KS, Gobet F. Video game training does not enhance cognitive ability: A comprehensive meta-analytic investigation. Psychol Bull. 2018;144: 111–139. doi: 10.1037/bul0000139 29239631

[65] Dye MWG, Bavelier D. Differential development of visual attention skills in school-age children. Vision Research. 2010;50: 452–459. doi: 10.1016/j.visres.2009.10.010 19836409 PMC2824025

[66] Franceschini S, Gori S, Ruffino M, Viola S, Molteni M, Facoetti A. Action Video Games Make Dyslexic Children Read Better. Current Biology. 2013;23: 462–466. doi: 10.1016/j.cub.2013.01.044 23453956

[67] Franceschini S, Trevisan P, Ronconi L, Bertoni S, Colmar S, Double K, et al. Action video games improve reading abilities and visual-to-auditory attentional shifting in English-speaking children with dyslexia. Sci Rep. 2017;7. doi: 10.1038/s41598-017-05826-8 28725022 PMC5517521

[68] Cardoso-Leite P, Joessel A, Bavelier D. Games for enhancing cognitive abilities. In: Plass J, Mayer RE, Homer BD, editors. Handbook of Game-based Learning. Boston: MIT Press; 2020. Available: https://mitpress.mit.edu/books/handbook-game-based-learning

[69] Pujol J, Fenoll R, Forns J, Harrison BJ, Martinez-Vilavella G, Macià D, et al. Video gaming in school children: How much is enough? Ann Neurol. 2016;80: 424–433. doi: 10.1002/ana.24745 27463843

[70] Przybylski AK. Electronic Gaming and Psychosocial Adjustment. Pediatrics. 2014;134: e716–e722. doi: 10.1542/peds.2013-4021 25092934

[71] Kovess-Masfety V, Keyes K, Hamilton A, Hanson G, Bitfoi A, Golitz D, et al. Is time spent playing video games associated with mental health, cognitive and social skills in young children? Soc Psychiatry Psychiatr Epidemiol. 2016;51: 349–357. doi: 10.1007/s00127-016-1179-6 26846228 PMC4814321

[72] Posso A. Internet usage and educational outcomes among 15-year old Australian students. International Journal of Communication. 2016;10: 26.

[73] Ferguson CJ. Do Angry Birds Make for Angry Children? A Meta-Analysis of Video Game Influences on Children’s and Adolescents’ Aggression, Mental Health, Prosocial Behavior, and Academic Performance. Perspect Psychol Sci. 2015;10: 646–666. doi: 10.1177/1745691615592234 26386002

[74] Hartanto A, Toh WX, Yang H. Context counts: The different implications of weekday and weekend video gaming for academic performance in mathematics, reading, and science. Computers & Education. 2018;120: 51–63. doi: 10.1016/j.compedu.2017.12.007

[75] Drummond A, Sauer JD. Timesplitters: Playing video games before (but not after) school on weekdays is associated with poorer adolescent academic performance. A test of competing theoretical accounts. Computers & Education. 2020;144: 103704. doi: 10.1016/j.compedu.2019.103704

[76] Ventura M, Shute V, Kim YJ. Video gameplay, personality and academic performance. Computers & Education. 2012;58: 1260–1266. doi: 10.1016/j.compedu.2011.11.022

[77] Cardoso-Leite P, Bavelier D. Video game play, attention, and learning: How to shape the development of attention and influence learning? Current Opinion in Neurology. 2014;27: 185–191. doi: 10.1097/WCO.0000000000000077 24553464

[78] Libertus ME, Liu A, Pikul O, Jacques T, Cardoso-Leite P, Halberda J, et al. The Impact of Action Video Game Training on Mathematical Abilities in Adults. AERA Open. 2017;3: 233285841774085. doi: 10.1177/2332858417740857

[79] Parry DA, le Roux DB. Media multitasking and cognitive control: A systematic review of interventions. Computers in Human Behavior. 2019;92: 316–327. doi: 10.1016/j.chb.2018.11.031

[80] Clifford S, Doane LD, Breitenstein R, Grimm KJ, Lemery-Chalfant K. Effortful Control Moderates the Relation Between Electronic-Media Use and Objective Sleep Indicators in Childhood. Psychol Sci. SAGE Publications Inc; 2020;31: 822–834. doi: 10.1177/0956797620919432 32558622 PMC7492726

[81] Lange K, Cohrs S, Skarupke C, Görke M, Szagun B, Schlack R. Electronic media use and insomnia complaints in German adolescents: Gender differences in use patterns and sleep problems. J Neural Transm. 2017;124: 79–87. doi: 10.1007/s00702-015-1482-5 26577762

[82] Stiglic N, Viner RM. Effects of screentime on the health and well-being of children and adolescents: A systematic review of reviews. BMJ Open. 2019;9: e023191. doi: 10.1136/bmjopen-2018-023191 30606703 PMC6326346

[83] Borsboom D, Cramer AOJ. Network Analysis: An Integrative Approach to the Structure of Psychopathology. Annual Review of Clinical Psychology. 2013;9: 91–121. doi: 10.1146/annurev-clinpsy-050212-185608 23537483

[84] Epskamp S, Borsboom D, Fried EI. Estimating psychological networks and their accuracy: A tutorial paper. Behavior Research Methods. 2018;50: 195–212. doi: 10.3758/s13428-017-0862-1 28342071 PMC5809547

[85] Diamond A. Executive functions. Annu Rev Psychol. 2013;64: 135–168. doi: 10.1146/annurev-psych-113011-143750 23020641 PMC4084861

[86] School grade placement. Stamford American International School Singapore. 2019; Available: https://www.sais.edu.sg/admissions/grade-listings/

[87] Conners C. Conners’ Rating Scales-Revised technical manual. North Tonawanda, NY: Multi-Health Systems; 1997.

[88] Mrazek MD, Phillips DT, Franklin MS, Broadway JM, Schooler JW. Young and restless: Validation of the Mind-Wandering Questionnaire (MWQ) reveals disruptive impact of mind-wandering for youth. Frontiers in Psychology. 2013;4. doi: 10.3389/fpsyg.2013.00560 PMC3753539 23986739

[89] Kessler RC, Barker PR, Colpe LJ, Epstein JF, Gfroerer JC, Hiripi E, et al. Screening for serious mental illness in the general population. Arch Gen Psychiatry. 2003;60: 184–189. doi: 10.1001/archpsyc.60.2.184 12578436

[90] Dweck CS. Mindset: The new psychology of success. Ballantine Books trade pbk. ed. New York: Ballantine Books; 2008.

[91] Brickenkamp R, Zillmer E. The D2 test of attention. Hogrefe & Huber Seattle, WA; 1998.

[92] Robertson IH, Manly T, Andrade J, Baddeley BT, Yiend J. ‘Oops!’: Performance correlates of everyday attentional failures in traumatic brain injured and normal subjects. Neuropsychologia. 1997;35: 747–758. doi: 10.1016/S0028-3932(97)00015-8 9204482

[93] Petton M, Perrone-Bertolotti M, Mac-Auliffe D, Bertrand O, Aguera P-E, Sipp F, et al. BLAST: A short computerized test to measure the ability to stay on task. Normative behavioral data and detailed cortical dynamics. bioRxiv. 2018. doi: 10.1101/498691 31541659

[94] R Core Team. R: A language and environment for statistical computing [Internet]. Vienna, Austria: R Foundation for Statistical Computing; 2019. Available: https://www.R-project.org/

[95] Wickham H, Averick M, Bryan J, Chang W, McGowan LD, Franà§ois R, et al. Welcome to the Tidyverse. Journal of Open Source Software. 2019;4: 1686. doi: 10.21105/joss.01686

[96] Bates D, Mächler M, Bolker B, Walker S. Fitting Linear Mixed-Effects Models Using Lme4. J Stat Soft. 2015;67. doi: 10.18637/jss.v067.i01

[97] Kuznetsova A, Brockhoff PB, Christensen RHB. lmerTest Package: Tests in Linear Mixed Effects Models. J Stat Soft. 2017;82. doi: 10.18637/jss.v082.i13

[98] Team RC. R: A language and environment for statistical computing. 2013

[99] Cotten SR, Shank DB, Anderson WA. Gender, technology use and ownership, and media-based multitasking among middle school students. Computers in Human Behavior. 2014;35: 99–106. doi: 10.1016/j.chb.2014.02.041

[100] Bavelier D, Green CS, Dye MWG. Children, Wired: For Better and for Worse. Neuron. 2010;67: 692–701. doi: 10.1016/j.neuron.2010.08.035 20826302 PMC3170902

[101] Reilly D, Neumann DL, Andrews G. Gender Differences in Spatial Ability: Implications for STEM Education and Approaches to Reducing the Gender Gap for Parents and Educators. In: Khine MS, editor. Visual-spatial Ability in STEM Education: Transforming Research into Practice. Cham: Springer International Publishing; 2017. pp. 195–224. 10.1007/978-3-319-44385-0_10

[102] Uzun AM, Kilis S. Does persistent involvement in media and technology lead to lower academic performance? Evaluating media and technology use in relation to multitasking, self-regulation and academic performance. Computers in Human Behavior. 2019;90: 196–203. doi: 10.1016/j.chb.2018.08.045

[103] Chaput J-P, Gray CE, Poitras VJ, Carson V, Gruber R, Olds T, et al. Systematic review of the relationships between sleep duration and health indicators in school-aged children and youth. Appl Physiol Nutr Metab. 2016;41: S266–S282. doi: 10.1139/apnm-2015-0627 27306433

[104] Owens J, ADOLESCENT SLEEP WORKING GROUP, COMMITTEE ON ADOLESCENCE. Insufficient Sleep in Adolescents and Young Adults: An Update on Causes and Consequences. PEDIATRICS. 2014;134: e921–e932. doi: 10.1542/peds.2014-1696 25157012 PMC8194472

[105] Stevens C, Bavelier D. The role of selective attention on academic foundations: A cognitive neuroscience perspective. Developmental Cognitive Neuroscience. 2012;2: S30–S48. doi: 10.1016/j.dcn.2011.11.001 22682909 PMC3375497

[106] Weis R, Cerankosky BC. Effects of Video-Game Ownership on Young Boys’ Academic and Behavioral Functioning: A Randomized, Controlled Study. Psychol Sci. SAGE Publications Inc; 2010;21: 463–470. doi: 10.1177/0956797610362670 20424084

[107] Salkind NJ, Wright JC. The development of reflection-impulsivity and cognitive efficiency. Human development. Karger Publishers; 1977;20: 377–387.

[108] Conard MA. Aptitude is not enough: How personality and behavior predict academic performance. Journal of Research in Personality. 2006;40: 339–346. doi: 10.1016/j.jrp.2004.10.003

[109] Bruni O, Ferinistrambi L, Russo P, Antignani M, Innocenzi M, Ottaviano P, et al. Sleep disturbances and teacher ratings of school achievement and temperament in children. Sleep Medicine. 2006;7: 43–48. doi: 10.1016/j.sleep.2005.09.003 16309959

[110] Dyson MP, Hartling L, Shulhan J, Chisholm A, Milne A, Sundar P, et al. A Systematic Review of Social Media Use to Discuss and View Deliberate Self-Harm Acts. Seedat S, editor. PLOS ONE. 2016;11: e0155813. doi: 10.1371/journal.pone.0155813 27191728 PMC4871432

[111] Yi Lin L, Sidani JE, Shensa A, Radovic A, Miller E, Colditz JB, et al. ASSOCIATION BETWEEN SOCIAL MEDIA USE AND DEPRESSION AMONG U.S. YOUNG ADULTS: Research Article: Social Media and Depression. Depression and Anxiety. 2016;33: 323–331. doi: 10.1002/da.22466 26783723 PMC4853817

[112] Lobel A, Engels RCME, Stone LL, Granic I. Gaining a competitive edge: Longitudinal associations between children’s competitive video game playing, conduct problems, peer relations, and prosocial behavior. Psychology of Popular Media Culture. 2017. doi: 10.1037/ppm0000159

[113] Hampton KN. Studying the Digital: Directions and Challenges for Digital Methods. Annual Review of Sociology. 2017;43: 167–188. doi: 10.1146/annurev-soc-060116-053505

[114] Rich M, Bickham DS, Shrier LA. Measuring Youth Media Exposure: A Multimodal Method for Investigating the Influence of Media on Digital Natives. American Behavioral Scientist. 2015;59: 1736–1754. doi: 10.1177/0002764215596558

[115] Greenberg BS, Eastin MS, Skalski P, Cooper L, Levy M, Lachlan K. Comparing Survey and Diary Measures of Internet and Traditional Media Use. Communication Reports. 2005;18: 1–8. doi: 10.1080/08934210500084164

[116] Scharkow M. The Accuracy of Self-Reported Internet Use—A Validation Study Using Client Log Data. Communication Methods and Measures. 2016;10: 13–27. doi: 10.1080/19312458.2015.1118446

