# Supplementary material for: Media use, attention, mental health and academic performance among 8 to 12 year old children
Source: PLoS One. 2021 Nov 17;16(11):e0259163. doi: 10.1371/journal.pone.0259163 (PMC8598050; doi:10.1371/journal.pone.0259163)
Supplement: S1 File — This file contains additional information about the methods and dataset as well as supplementary analyses. (PDF) [file pone.0259163.s001.pdf]

Supplementary Materials

27/10/2021

Contents

1 Methods **2**

1.1 Surveys . . . 2

1.2 Strength and Difficulties questionnaire . . . 3

1.3 Cognitive Tasks . . . 5

1.4 Procedure . . . 6

2 Analyses **7**

2.1 Reliability . . . 7

2.2 Supplementary figures . . . 7

2.3 Partial correlations . . . 10

3 Codebook **12**

4 R session information **15**

References **16**

1 Methods

1.1 Surveys

1.1.1 Technology Usage Questionnaire

1.1.1.1 Media multitasking inventory This questionnaire is adapted from the Media Use Questionnaire developed by Ophir and colleagues [1]. It comprises two parts. The first part asks participants to rate how much time they spend on various media uses separately for week days and weekend days. The eight media uses considered were

1. reading (on paper or screen),
2. watching videos (films, TV, YouTube),
3. listening to music,
4. writing (with pen, keyboard or other),
5. making images (drawing, photography, film) or music,
6. playing video games,
7. searching information on the internet,
8. discussing over the internet or telephone

Note: following Pea et al. [2] we also asked about the time spent talking with other people without using technology and did not include this data in the media-related scores.

For each of the 8 media, participants reported a frequency of use on a 5-point Likert scale: "Never," "Less than 1 hour per day," "1 to 2 hours per day," "2 to 4 hours per day," "More than 4 hours per day." Responses were then converted to numerical values (e.g., "Less than 1 hour per day" => 0.5 hours, "More than 4 hours per day" => 4.5 hours). We then computed the average time on each medium using a weighted sum (i.e., multiplying the reported number of hours during week days by 5/7 and the number of hours per weekend day by 2/7); the total number of hours of media content consumed per day is defined as the sum of these weighted averages across media. Note that we also extracted from this questionnaire the self-reported number of hours of daily video game play. This number of hours of video game play was used both in isolation (e.g., for the network analysis reported in the paper) and in combination with the data from the gaming questionnaire to estimate how many hours children spend on various game genres.

The second part of this questionnaire asks about the simultaneous use of multiple media (i.e., media multitasking). An example question would be "while you are reading, how often do you also watch videos?" to which participants reported either "Never," "Sometimes" or "Often." Although we asked about all combinations of media, we did not ask the questions in both directions (i.e., "while doing i, how often do you do j?" but not "while doing j, how often do you do i?"; below, we refer to these ratings as $r_{i,j}$ and assume $r_{i,j} = r_{j,i}$). This has the disadvantage of a reduced reliability but the advantage that it is less boring and time consuming.

As in Ophir and colleagues [1] we converted the reported frequencies of joint media uses to numeric values ("Never" = 0, "Sometimes" = 0.5 and "Often" = 1) and together with the number of hours per medium extracted from the first part of the questionnaire, we computed the media multitasking index, which reflects the number of additional media a person uses when using a medium. More specifically,

$$MMI = \sum_{i=1}^{8} \frac{m_i \times h_i}{h_{total}}$$

where

- m_i is the number of media used while using medium i, computed by summing the reported frequencies of simultaneously used media pairs (i.e., $m_i = \sum_{j \neq i} r_{i,j}$);

- h_i is the number of hours spent on medium i;
- $h_{total} = \sum_{i=1}^{8} h_i$ is the total time spent on media.

Note that the current questionnaire is not exactly the same as the one used by Ophir and colleagues [1]; as a result, the scores computed with our survey cannot be directly compared; they are however expected to be highly correlated [3].

In the current study we used three main measures from this questionnaire. The total number of hours of media content consumed per day, the media multitasking index (as defined in our study) and the total number of hours of video gaming per day

1.1.1.2 Video game play This questionnaire asks about which video games children play, on what device and how frequently ("often," "sometimes," "rarely"). An example response could be "Mario Kart" on "Nintendo DS" that is played "A lot." The names of the video games provided by the children were then entered into various online databases (e.g., http://www.esrb.org) to collect metadata and to classify the games according to various attributes (by two different experimenters; any discrepancy in classification was arbitrated by C.S. Green). The only attribute considered here was whether a game was classified as having mechanics from the action video game genre (which we call "action-like") or not ("other"). For example, *Call of Duty* and *GTA* were classified as "action-like" while *Angry Birds* and *Candy Crush* were classified as "other."

We used the total time spent on video games from the media multitasking questionnaire (e.g., 1 hour per day) and split that number into an estimate of how much time was spent on action-like versus other types of games (e.g., 45 minutes versus 15 minutes). To get an estimate of the time spent per type of game ("action-like" versus "other") we used the following procedure. For each of the games a participant reported playing, we converted the reported frequency of play into a numeric value (i.e., "often"= 1, "sometimes" = 0.66, "rarely" = 0.33) and then used those numbers to estimate the fraction of gaming time spend on that specific game. For example, if a child reported playing *Game A* "sometimes" (=0.66), *Game B* "sometimes" (=0.66) and *Game C* "often" (=1) we would estimate that the child spend 28.4% of their video gaming time on *Game A* (i.e., 0.66/(0.66 + 0.66 + 1) * 100), 28.4% on *Game B* and the remaining 43.1% (i.e., 1 / (0.66 + 0.66 + 1) * 100) on *Game C*. Next we estimate the percentage of time spent on action-like versus other games by summing the percentages within the game category. Continuing with our example, if *Game A* and *Game C* are both "action-like," while *Game B* is "other" we would evaluate that 71.6% (i.e., 28.4% + 43.1%) of the child's gaming time is spent on action-like games while the remainder 28.4% of gaming time is spent on "other" games. Finally, to get a sense of how much time is spent on each game category we split the reported total time on video games (e.g., 1 hour per day) following those percentages. In our example, this would translate into 43 minutes of playing "action-like" games (i.e., 71.6% of 1 hour) and 17 minutes of playing "other" games per day.

1.1.2 K-6 Distress scale

This questionnaire [4] evaluates non-specific psychological distress with 6 items relating to anxiety and depression (e.g., "During the past 30 days, about how often did you feel hopeless?"). A higher score reflects higher levels of distress.

1.2 Strength and Difficulties questionnaire

This 25 items questionnaire covers 5 dimensions (emotional symptoms, conduct problems, hyperactivity/inattention, peer relationship problems and prosocial behavior) and provides a total score with higher scores reflecting more general difficulties, encompassing both emotional and behavioral problems [5].

1.2.1 Conners Teacher's Rating Scale

This scale asks teachers to evaluate children's school behavior through 28 items [e.g., "inattentive, easily distracted" with response on a 4-point Likert scale going from "Not at all" to "Very much true"; [6]]. The scale can be used to compute various sub scores (e.g., "hyperactivity," "conduct problems"); for the purpose of the present study we only used the total score, with higher values indicating greater attentional/behavioral problems.

1.2.2 Conners Parent's Rating Scale

This scale asks parents to evaluate their child's behavior and personality through 48 items (e.g., "excitable, impulsive") with responses on a 4-point Likert scale going from "Not at all" to "Very much true" [7]. As with the teachers' version, the scale can be used to compute various sub scores (e.g., "impulsive-hyperactive," "conduct problems"). Again, for the purpose of this study we only used the total score, where higher values indicate greater attentional/behavioral problems.

1.2.3 Sleep Questionnaire

This custom-made questionnaire consists of 7 questions regarding the quality of sleep and the overall level of fatigue during the past month and 2 questions about the frequency of dreams of nightmares. Participants were asked to report how often during the last month they felt assertions like "I have not slept enough" or "I had a hard time waking up in the morning" by selecting among the response options "Never," "Sometimes" and "Often." We did not ask separately for week days and weekend days. The responses to these questions were converted to a numeric value ("Never" = 0, "Sometimes" = 0.5, "Often" = 1; or the opposite for items phrased in the opposite way) and averaged these values such that a higher score indicates better sleep and less fatigue. Only the data from the 7 sleep questions were included in this study. The greater the score the better the sleep.

1.2.4 Mind-wandering Questionnaire

This 5-item short survey [8] was used to assess the tendency for thoughts to drift (e.g., "I have difficulty maintaining focus on simple or repetitive work" rated on 5-point Likert scale). A higher score reflects a greater tendency to mind-wander.

1.2.5 Grit Questionnaire

The Grit questionnaire measures perseverance and passion for long-term goals [9]. It asks people to rate 12 statements (e.g., "I finish whatever I begin") on a 5-point Likert scale (ranging from "Very much like me" to "Not like me at all"). A higher score means greater grit.

1.2.6 Theory of Intelligence (growth versus fixed Mindset)

The questionnaire developed by Dweck and colleagues consists of 20 statements assessing people believes about the plasticity of their intelligence (e.g., "No matter how much intelligence you have, you can always change it quite a bit" rated on a 4-point Likert scale; [10]). Responses are used to compute a global score with higher scores indicating a greater belief that intelligence can be changed (i.e., a "growth" mindset, as opposed to a "fixed" mindset).

1.2.7 Grades and Demographics

This custom-made questionnaire asked about birth date, gender, handedness, number of siblings, self-reported health state, the languages spoken at home, as well as yes/no questions about difficulties in vision, audition, learning and verbal comprehension and expression. In addition, it also asks about self-reported grades (the question translates to "what do you think is your average general grade at school?"), and grade satisfaction ("I have good grades at school" with responses on a four point Likert scale going from yes to no). The main variables we took from this set of questions are age (calculated using the birth date at the moment of the first in-class test session in early January), gender and self-reported grade (grade satisfaction was not included).

1.2.8 Other questionnaires

Parents and children were given other questionnaires, which will not be reported here. These included an exploratory, custom-made "IAM" questionnaire containing 7 statements about one self to be rated (e.g., "I'm a fast learner," "I get easily bored"), a survey on extracurricular activities ("Do you have activities outside of school? Write below which activities you do, how often per week and how long each time it takes. For example: Football, twice per week, 1 hour per session."), and the number of devices in the child's room.

1.3 Cognitive Tasks

Children in this study were asked to complete 5 different cognitive tasks, tapping mostly into attentional processes. Out of these 5 tasks, only three are reported here: the D2, the Sustained Attention to Response Task, and the Bron Lyon Attention Stability Task. The two tasks not reported are the Multiple Object Tracking task (because it did not work properly) and a new statistical learning task (as it was part of a pilot for another project).

1.3.1 D2 cancellation task

The D2 task is a paper and pencil task designed to measure selective attention [11]. Participants were given a sheet of paper comprising 14 lines of symbols, each symbol comprising the letter "d" or "p" with zero, one or two bars above and/or below the letter. Following previous studies we used only the first 10 rows of the test [12]. Children were orally instructed to scan each line from left to right, symbol by symbol, and to circle every target: the letter "d" surrounded by exactly two bars (e.g., one above and one below, two above and none below, etc.). They were instructed to do so as quickly as possible while making as few errors as possible within the allocated time of 20 seconds per line. Each time the experimenter announced the end of the 20 seconds dedicated to a given line, participants were to stop the task and to draw a bar after the last symbol they processed in that line. After a couple of seconds, the experimenter announced the start time for the next line. The first row was used for training and to make sure every child understood the instructions and was familiar with the material. The subsequent nine rows were used for assessment.

As for all cognitive tasks in this work, we computed three measures from this task: a response speed index (the time needed to scan 1 item, i.e., 20 seconds divided by the average of the total number of items scanned per line), an inattention index (the rate of misses, i.e., target symbols that were not encircled) and an impulsivity index (the rate of false alarms, i.e., non-targets that were encircled).

1.3.2 Sustained Attention to Response Task (SART)

The SART task [13] was completed on digital tablets connected to the Internet. In this task a digit (1-9) appears on the screen center every 1'150 ms; it is displayed for 250 ms, followed by a 900 ms blank screen. The task instructs children to tap on the screen in response to any digit except the digit "3." All nine digits are presented equally often and hence on most trials children are to tap the screen and only on 1/9 of the trials are they to withhold responding. Children first went through a self-paced, step-by-step tutorial on the

tablet before doing the actual task. Children read the instructions on the screen and when needed were given further clarification by the experimenter. The task lasted for about four minutes.

As with the D2 task, we computed 3 indices from this task relating to response speed (median response time on correct trials), inattention (rate of misses, i.e., digits other the "3" that were not responded to) and impulsivity (rate of false alarms, i.e., digits "3" in response to which kids tapped the screen).

1.3.3 Bron Lyon Attention Stability Task (BLAST)

Children completed the Bron Lyon Attention Stability Task (BLAST) task [14] on digital tablets. They were first shown a single target letter (e.g., "A") for 250 ms, followed 500 ms later (ISI) by a 2x2 array of letters that did (e.g., "A, K, B, R") or did not (e.g., "X, K, B, R") contain the target letter. They were asked to press one of two buttons (drawn on the screen) to indicate whether or not the target letter was present in the array. Children were given a maximum time limit of 3 seconds after the onset of the array to respond. After each trial, they received visual feedback indicating whether their response was correct or not. If they failed to respond on time or responded incorrectly they would experience a timeout (i.e., forced pause) of 4'750 ms before the next trial.

Children first went through a self-paced, step-by-step tutorial on the tablet before doing the actual task. Children read the instructions on the screen and when needed were given further clarification by the experimenter. The task lasted for about four minutes.

As with the previous tests, we computed 3 indices from this task relating to response speed (median response time on correct trials), inattention (rate of misses, i.e., trials were kids report that the array did not contain target when in fact it did) and impulsivity (rate of false alarms, i.e., trials were kids report that the array did contain target when in fact it did not).

1.3.4 Composite scores of cognitive performance

To reduce the likelihood of spurious findings given the small sample size in this study and to possibly interpret results at the construct rather than at the measurement level we computed composite scores for speed, inattention and impulsivity by first z-scoring each of these variables within tasks before averaging them across tasks (e.g., the composite impulsivity score is the average of the z-scored impulsivity measures across the D2, the SART and the BLAST tasks). Finally, for response speed we multiplied the resulting score by -1 so that the measure would reflect response speed (higher values = faster) rather than response time.

1.4 Procedure

The teachers distributed and collected the consent forms from the parents at the beginning of the school year (September). Half of the questionnaires were distributed via the teachers to the parents who consented to participate in the study in December. A few months later the remainder of questionnaires was distributed. Table 1 lists the questionnaires included in each batch. Parents could fill out the questionnaires at home with their child and bring them back to the school once they were completed. We decided to split the questionnaires into two batches to be filled out a few months apart in order to favor higher completion rates given the large number of questionnaires used in this study. We expected more people to complete the first batch than the second batch of questionnaires. Teachers also filled out a questionnaire (Conners Teachers' Rating Scale) on each of their pupils whose parents had consented to the study.

Table 1. Surveys used in the study.

	survey
**Parents surveys: part 1**	general / demographics
	mindset

	survey
	sleep
	media (daily hours on each medium)
	media_multitasking: combination of pairs of media
	number of devices kids have in their room
	video games: report games, frequency of play and console
**Parent surveys: part 2**	Grit
	iam
	mind wandering
	Distress (K6)
	extracurricular activities
	strength and difficulties (SDQ)
	Conners ratings
**Teacher's survey**	Conners ratings

2 Analyses

2.1 Reliability

Table 2. Chronbach's alpha of the surveys used in this study.

Survey	number of questions	Chronbach's alpha
Distress (K6)	6	0.69
SDQ	25	0.81
Teacher ratings	28	0.95
Parent ratings	48	0.91
Sleep	7	0.69
Mind-wandering	5	0.72
Grit	12	0.68
Mindset	20	0.67

2.2 Supplementary figures

2.2.1 Total hours of media by gender

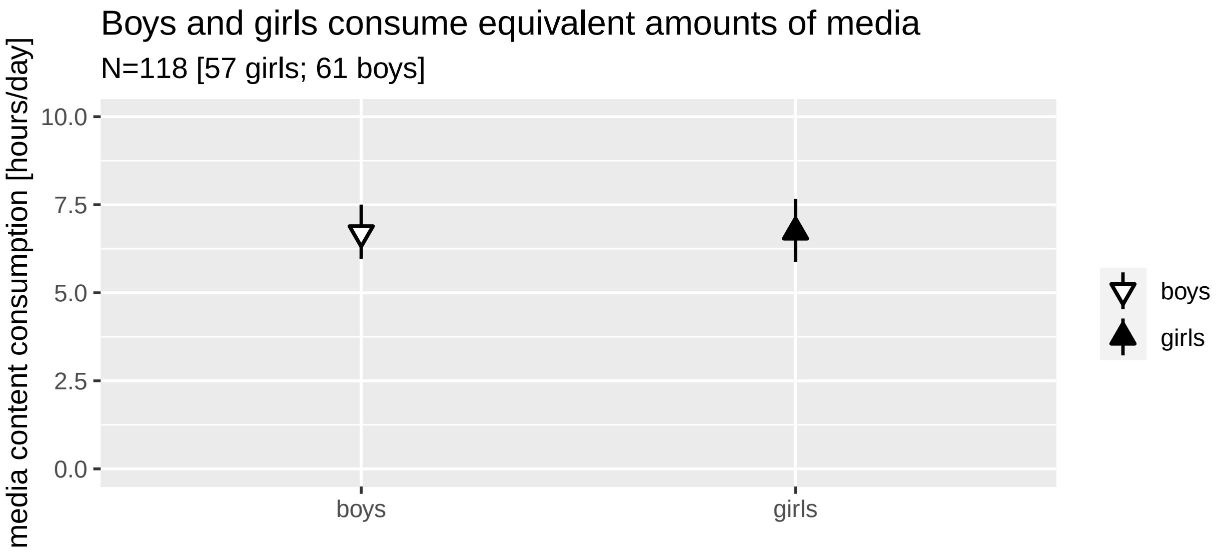

2.2.2 Daily hours boys and girls spent on each medium

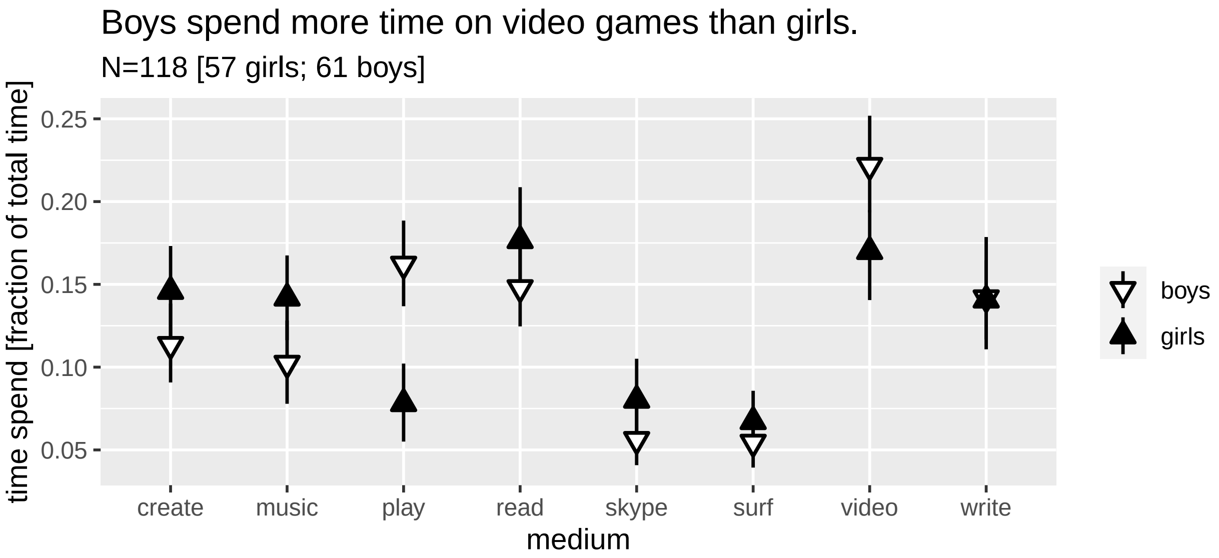

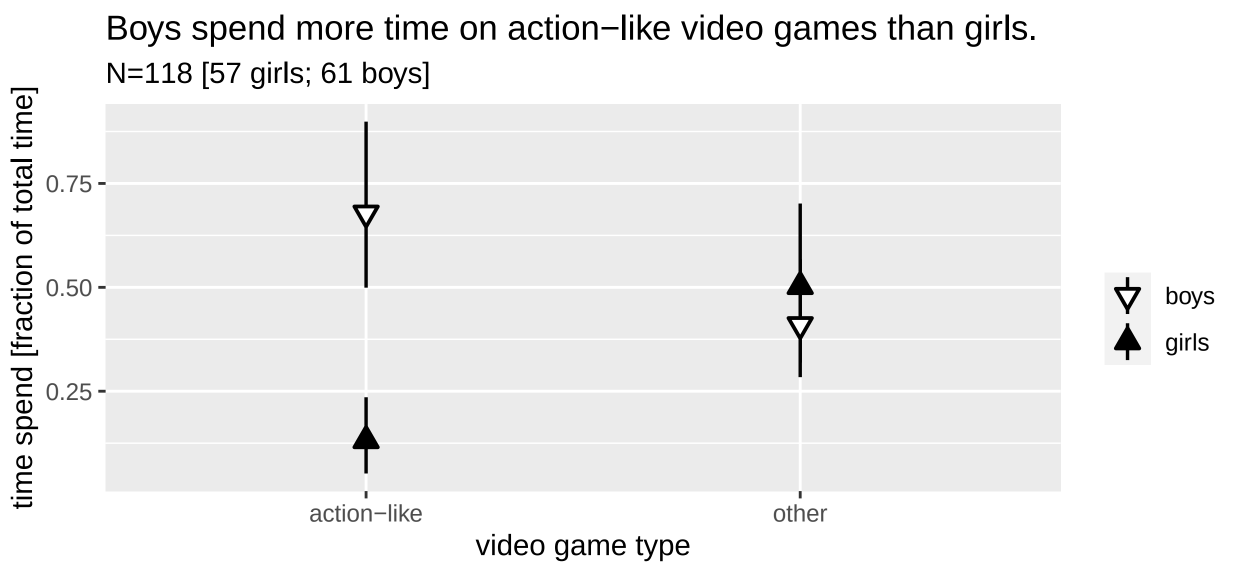

2.2.3 Media multitasking by age

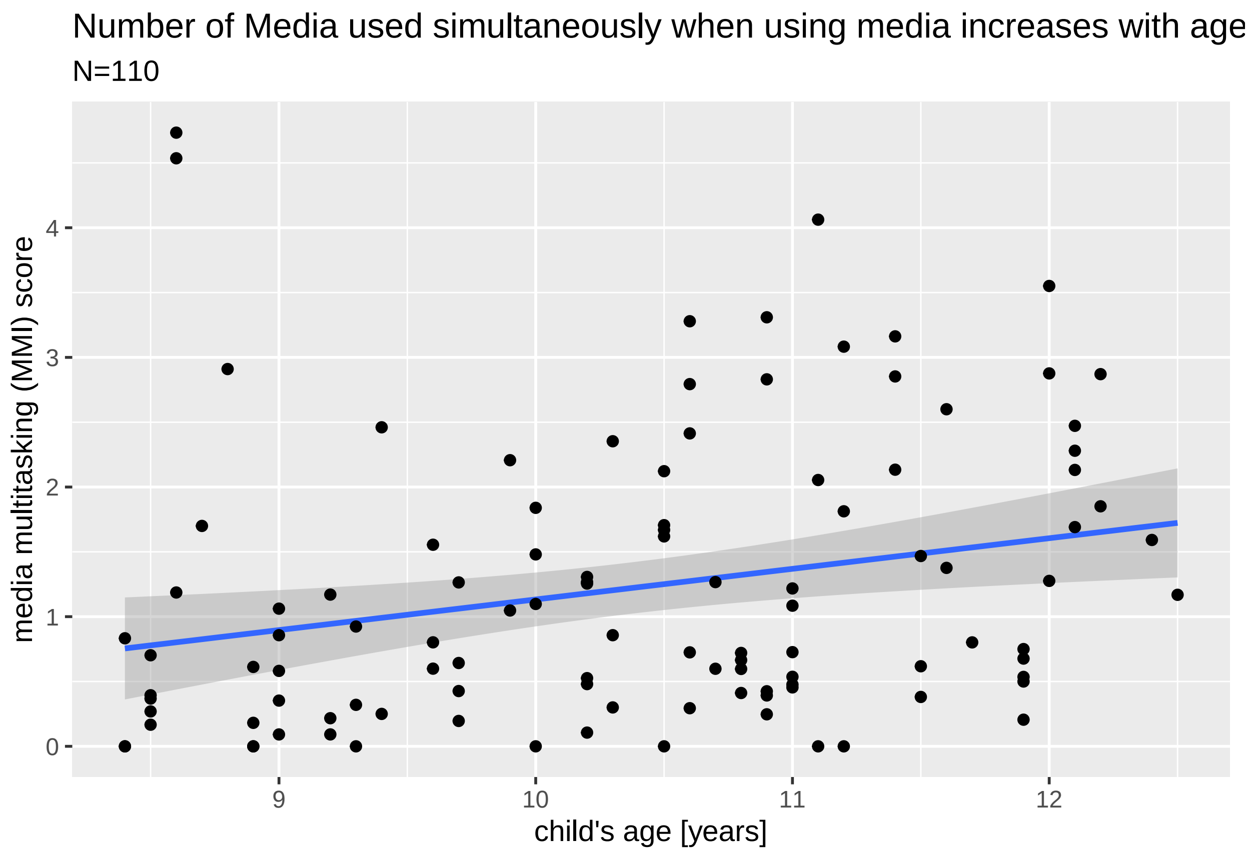

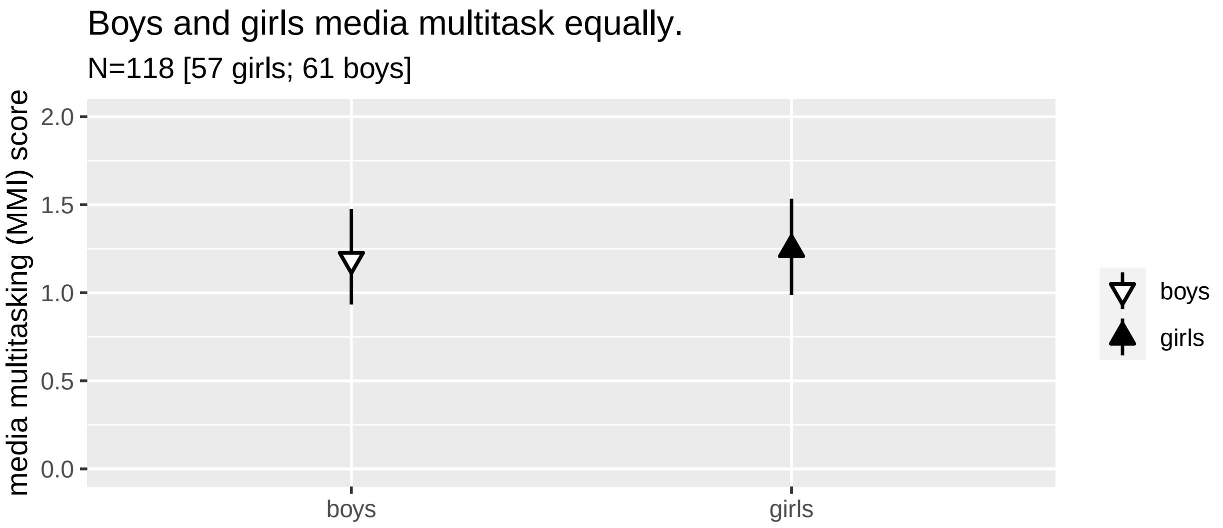

2.3 Partial correlations

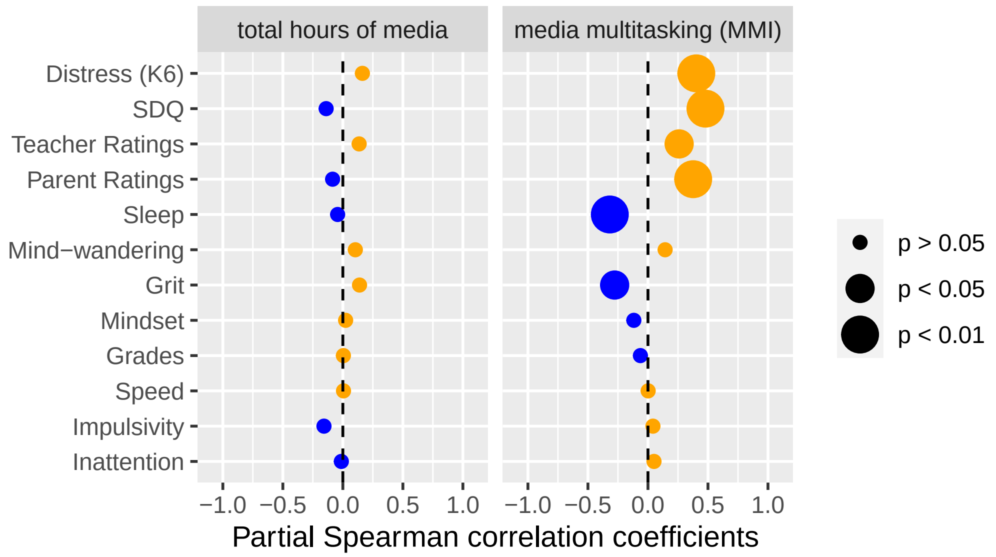

Figure 1: Partial Spearman correlation profiles of total hours of media (controlling for age, gender, time spent on video games and MMI; left panel) and media multitasking (MMI; controlling for age, gender, time spent on video games and total hours of media; right panel).

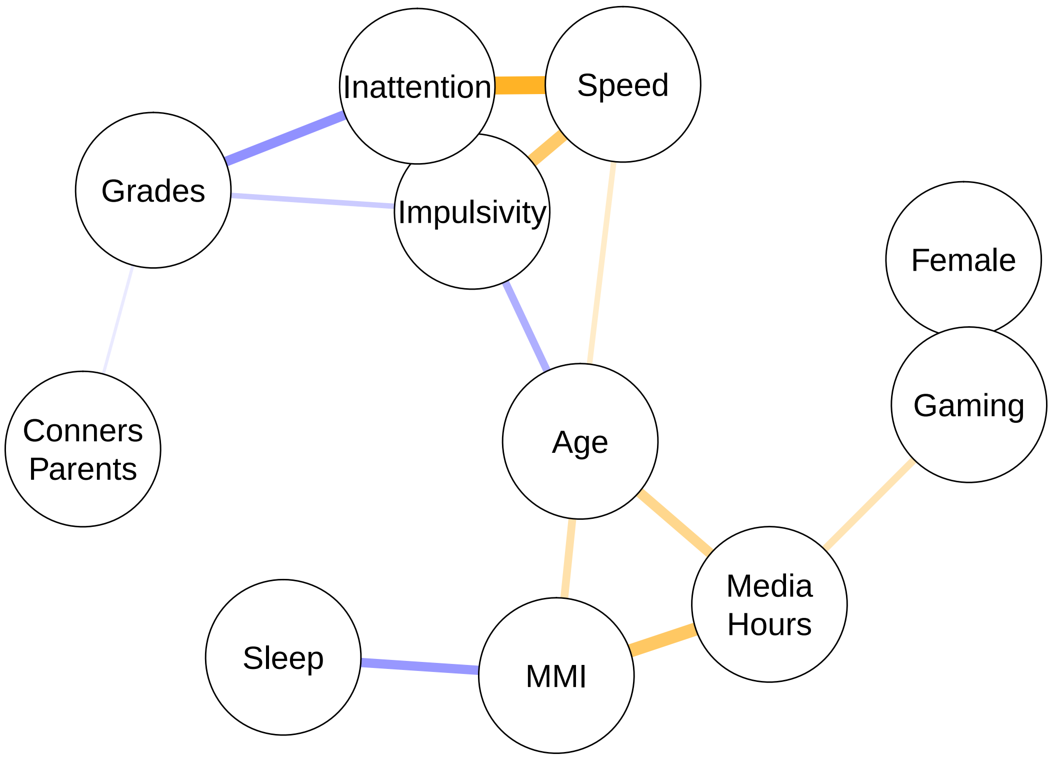

Figure 2: This Concentration Plot represents the network structure estimated on the 11 variables of interest. Nodes represent variables; edges represent the relationship between variables that cannot be explained by the remaining variables. The width and saturation of the edges reflect the strength of the relationship; positive associations are highlighted in orange and negative associations in blue (e.g., higher levels of media multitasking (MMI) are associated with being older, higher number of hours of media and worse sleep quality).

3 Codebook

This codebook refers to the dataset `mmi_kids_gva_v2021.2.csv` which is the basis for the study:

> Cardoso-Leite, P., Buchard, A., Tissieres, I., Mussack, D., & Bavelier, D. (2020, December 22). Media use, attention, mental health and academic performance among 8 to 12 year old children. https://doi.org/10.31234/osf.io/bhnam

This dataset comprises 156 rows (children) and 42 columns (variables). Missing values are coded `NA`. The text below lists and describes all the columns of that dataset; for further details see the paper and other supplementary material (https://osf.io/aj2bc/).

—— ID and context variables ——

kid [string]

uniquely identifies a child in the dataset (e.g., “k001”).

teacher [string]

uniquely identifies a teacher in the dataset (e.g., “t01”).

school_year [string]

the child’s grade level at the time of testing. Possible values are: - 5P - 6P - 7P - 8P - special needs

age [float]

age of the child expressed in year (e.g., 11.9).

age_group [string]

age of the child expressed as one of three age range (in years): - [8.0,10.2) - [10.2,11.2) - [11.2,12.7]

gender [string]

the child’s gender. - male - female

grades [float]

values from 0 to 1, reflecting the child’s self-reported average grades (0 = lowest possible grade, 1 = highest possible grade).

—— Media variables ——

read_h [float]

estimated number of daily hours the child spends reading.

video_h [float]

estimated number of daily hours the child spends watching videos.

music_h [float]

estimated number of daily hours the child spends listening to music.

write_h [float]

estimated number of daily hours the child spends writing.

create_h [float]

estimated number of daily hours the child spends creating content (e.g., images).

play_h [float]

estimated number of daily hours the child spends playing video games.

play_action_h [float]

estimated number of daily hours the child spends playing “action-like” video games. Note that some children might have reported the time spent playing video games without however specifying which kinds of game were played.

play_nonaction_h [float]

estimated number of daily hours the child spends playing video games that are not “action-like.” Note that some children might have reported the time spent playing video games without however specifying which kinds of game were played.

surf_h [float]

estimated number of daily hours the child spends searching information on the internet.

skype_h [float]

estimated number of daily hours the child spends talking to others using technology.

talk_h [float]

estimated number of daily hours the child spends talking to others without using technology.

total_hours_weekday [float]

estimated total number of daily hours the child spends on media during a week day (i.e., excluding weekend days).

total_hours_weekendday [float]

estimated total number of daily hours the child spends on media during a weekend day (i.e., Saturday or Sunday).

media_h [float]

estimated total number of daily hours the child spends on media; computed as:

```
media_h = read_h + video_h + music_h + create_h + write_h + play_h + surf_h + skype_h
```

mmi_score [float]

score on the media multitasking inventory which measures the average number of additional media used simultaneously when using a medium; higher scores indicate a larger number of simultaneously used media. Possible values range from 0 (media used only in single tasking mode) to 7 (using all possible media in the list, all the time; the list of media included in the mmi score computation are “reading,” “watching videos,” “listening to music,” “creating content,” “writing,” “playing video games,” “browsing the internet” and “talking with others using technology”).

mmi_week [float]

mmi score based on week days media consumption.

mmi_weekend [float]

mmi score based on weekend days media consumption.

mm_frequency [float]

self-reported frequency of using multiple media at the same time. Possible values range from 0 (media used only in single tasking mode) to 1 (always using more than one media at the same time, when using media).

—— Media variables ——

k6_score [float]

score on the K-6 distress scale which evaluates non-specific psychological distress with items relating to anxiety and depression; a higher score reflects higher levels of emotional distress.

sdq_score [float]

score on the Strength and Difficulties questionnaire; a higher score reflects more greater socio-emotional difficulties.

ct_score [float]

score reflects how teachers rate their pupils on specific dimensions; a higher score reflects overall more ADHD-like behavior (i.e., teacher perceives the child as having more difficulties paying attention or engaging more in impulsive behaviors).

cp_score [float]

score reflects how parents rate their child on specific dimensions; a higher score reflects overall more ADHD-like behavior (i.e., parent perceives the child as having more difficulties paying attention or engaging more in impulsive behaviors).

sleep_score [float]

estimate of the child’s sleep quality; a higher score indicates better sleep and less fatigue.

mw_score [float]

the child’s score on the mind wandering questionnaire; a higher score indicates the child has a higher frequency to experience task unrelated thoughts (i.e., mind wandering).

grit_score [float]

the child’s score on the grit questionnaire; higher scores reflect perseverance and passion for long-term goals.

toi_score [float]

the child's score on the theory of intelligence questionnaire (i.e., "mindset"); a higher score indicates a "growth mindset" or a stronger belief that intelligence can be improved.

—— Cognitive test variables ——

d2_rt [float]

average response time per processed symbol in the d2 cancellation test expressed in seconds.

d2_fa_rate [float]

average rate ([0-1]) at which a child incorrectly cancelled a symbol in the d2 cancellation test.

d2_miss_rate [float]

average rate ([0-1]) at which a child incorrectly failed to cancel a symbol in the d2 cancellation test.

sart_rt [float]

sart is a go/no-go task. This variable refers to the average response time on correct "go" trials only, expressed in seconds.

sart_fa_rate [float]

average rate ([0-1]) at which a child incorrectly responded ("go" response) in the sart test (a go/no-go test).

sart_miss_rate [float]

average rate ([0-1]) at which a child incorrectly failed to respond (i.e., "no-go" response) in the sart test (a go/no-go test).

blast_rt [float]

average response on correct trials only of the BLAST test, expressed in seconds.

blast_fa_rate [float]

average rate ([0-1]) at which a child incorrectly reported a stimulus array to contain a probe symbol.

blast_miss_rate [float]

average rate ([0-1]) at which a child incorrectly failed to report the presence of a probe symbol in the stimulus array.

4 R session information

The following describes the environment and packages used in this project.

- R version 3.6.0 (2019-04-26), `x86_64-apple-darwin15.6.0`

- Locale: `en_US.UTF-8/en_US.UTF-8/en_US.UTF-8/C/en_US.UTF-8/en_US.UTF-8`
- Running under: `macOS  10.15.7`
- Matrix products: default
- BLAS: `/Library/Frameworks/R.framework/Versions/3.6/Resources/lib/libRblas.0.dylib`
- LAPACK: `/Library/Frameworks/R.framework/Versions/3.6/Resources/lib/libRlapack.dylib`
- Base packages: base, datasets, graphics, grDevices, methods, stats, utils
- Other packages: bootnet 1.4.3, dplyr 1.0.6, forcats 0.5.0, ggplot2 3.3.3, lme4 1.1-26, lmerTest 3.1-3, Matrix 1.2-18, purrr 0.3.4, readr 1.4.0, stringr 1.4.0, tibble 3.1.2, tidyr 1.1.3, tidyverse 1.3.0
- Loaded via a namespace (and not attached): abind 1.4-5, assertthat 0.2.1, backports 1.2.1, base64enc 0.1-3, BDgraph 2.63, bookdown 0.21, boot 1.3-25, broom 0.7.2.9000, candisc 0.8-3, car 3.0-10, carData 3.0-4, cellranger 1.1.0, checkmate 2.0.0, class 7.3-17, cli 2.5.0, cluster 2.1.0, codetools 0.2-18, colorspace 2.0-1, compiler 3.6.0, corpcor 1.6.9, crayon 1.4.1, curl 4.3, d3Network 0.5.2.1, data.table 1.14.0, DBI 1.1.0, dbplyr 2.0.0, digest 0.6.27, doParallel 1.0.16, e1071 1.7-4, eigenmodel 1.11, ellipse 0.4.2, ellipsis 0.3.2, evaluate 0.14, fansi 0.4.2, fdrtool 1.2.15, foreach 1.5.1, foreign 0.8-75, Formula 1.2-4, fs 1.5.0, gdata 2.18.0, generics 0.1.0, glasso 1.11, glmnet 4.0-2, glue 1.4.2, grid 3.6.0, gridExtra 2.3, gtable 0.3.0, gtools 3.8.2, haven 2.3.1, heplots 1.3-7, Hmisc 4.4-2, hms 0.5.3, htmlTable 2.2.1, htmltools 0.5.1.1, htmlwidgets 1.5.3, httr 1.4.2, huge 1.3.4.1, igraph 1.2.6, IsingFit 0.3.1, IsingSampler 0.2.1, iterators 1.0.13, jpeg 0.1-8.1, jsonlite 1.7.2, knitr 1.33, lattice 0.20-41, latticeExtra 0.6-29, lavaan 0.6-8, lifecycle 1.0.0, lubridate 1.7.9.2, magrittr 2.0.1, MASS 7.3-53, matrixcalc 1.0-3, mgm 1.2-10, mice 3.12.0, minqa 1.2.4, mitools 2.4, mnormt 2.0.2, modelr 0.1.8, munsell 0.5.0, mvtnorm 1.1-1, NetworkToolbox 1.4.1, networktools 1.2.3, nlme 3.1-151, nloptr 1.2.2.2, nnet 7.3-14, nnls 1.4, numDeriv 2016.8-1.1, openxlsx 4.2.3, parallel 3.6.0, pbapply 1.4-3, pbivnorm 0.6.0, pillar 1.6.1, pkgconfig 2.0.3, plotrix 3.7-8, plyr 1.8.6, png 0.1-7, polynom 1.4-0, psych 2.0.12, qgraph 1.6.5, R.methodsS3 1.8.1, R.oo 1.24.0, R.utils 2.10.1, R6 2.5.0, RColorBrewer 1.1-2, Rcpp 1.0.5, readxl 1.3.1, relaimpo 2.2-3, reprex 0.3.0, reshape2 1.4.4, rio 0.5.16, rjson 0.2.20, rlang 0.4.11, rmarkdown 2.6, rpart 4.1-15, rstudioapi 0.13, rticles 0.18, rvest 0.3.6, scales 1.1.1, shape 1.4.5, smacof 2.1-1, splines 3.6.0, statmod 1.4.35, stats4 3.6.0, stringi 1.6.2, survey 4.0, survival 3.2-7, tidyselect 1.1.1, tmvnsim 1.0-2, tools 3.6.0, utf8 1.2.1, vctrs 0.3.8, weights 1.0.1, whisker 0.4, withr 2.4.2, wordcloud 2.6, xfun 0.24, xml2 1.3.2, yaml 2.2.1, zip 2.1.1

References

1. Ophir E, Nass C, Wagner AD. Cognitive control in media multitaskers. PNAS. 2009;106: 15583–7. doi:10.1073/pnas.0903620106

2. Pea R, Nass C, Meheula L, Rance M, Kumar A, Bamford H, et al. Media use, face-to-face communication, media multitasking, and social well-being among 8- to 12-year-old girls. Developmental Psychology. 2012;48: 327–336. doi:10.1037/a0027030

3. Baumgartner SE, Lemmens JS, Weeda WD, Huizinga M. Measuring Media Multitasking: Development of a Short Measure of Media Multitasking for Adolescents. Journal of Media Psychology. 2016; 1–10. doi:10.1027/1864-1105/a000167

4. Kessler RC, Barker PR, Colpe LJ, Epstein JF, Gfroerer JC, Hiripi E, et al. Screening for serious mental illness in the general population. Arch Gen Psychiatry. 2003;60: 184–189.

5. Goodman R. The Strengths and Difficulties Questionnaire: A research note. J Child Psychol Psychiatry. 1997;38: 581–586.

6. Conners C. Conners' Rating Scales-Revised technical manual. North Tonawanda, NY: Multi-Health Systems; 1997.

7. Goyette CH, Conners CK, Ulrich RF. Normative data on revised Conners Parent and Teacher Rating Scales. J Abnorm Child Psychol. 1978;6: 221–236.

8. Mrazek MD, Phillips DT, Franklin MS, Broadway JM, Schooler JW. Young and restless: Validation of the Mind-Wandering Questionnaire (MWQ) reveals disruptive impact of mind-wandering for youth. Frontiers in Psychology. 2013;4. doi:10.3389/fpsyg.2013.00560

9. Duckworth AL, Peterson C, Matthews MD, Kelly DR. Grit: Perseverance and passion for long-term goals. Journal of Personality and Social Psychology. 2007;92: 1087–1101. doi:10.1037/0022-3514.92.6.1087

10. Dweck CS. Mindset: The new psychology of success. Ballantine Books trade pbk. ed. New York: Ballantine Books; 2008.

11. Brickenkamp R, Zillmer E. The D2 test of attention. Hogrefe & Huber Seattle, WA; 1998.

12. de Ribaupierre A, Lecerf T. Relationships between working memory and intelligence from a developmental perspective: Convergent evidence from a neo-Piagetian and a psychometric approach. European Journal of Cognitive Psychology. 2006;18: 109–137. doi:10.1080/09541440500216127

13. Robertson IH, Manly T, Andrade J, Baddeley BT, Yiend J. 'Oops!': Performance correlates of everyday attentional failures in traumatic brain injured and normal subjects. Neuropsychologia. 1997;35: 747–758. doi:10.1016/S0028-3932(97)00015-8

14. Petton M, Perrone-Bertolotti M, Mac-Auliffe D, Bertrand O, Aguera P-E, Sipp F, et al. BLAST : A short computerized test to measure the ability to stay on task. Normative behavioral data and detailed cortical dynamics. bioRxiv. 2018; doi:10.1101/498691
